# The Role of the Organic Moiety in the Diffusion and Transport of Carboxylates into Liposomes

**DOI:** 10.3390/molecules29215124

**Published:** 2024-10-30

**Authors:** Aaron Torres-Huerta, Hennie Valkenier

**Affiliations:** Engineering of Molecular NanoSystems, Ecole Polytechnique de Bruxelles, Université Libre de Bruxelles (ULB), Avenue F. Roosevelt 50, CP165/64, B-1050 Brussels, Belgium; aaron.torres.huerta@ulb.be

**Keywords:** supramolecular chemistry, anion transport, transmembrane transport, carboxylates, membrane diffusion, liposomes

## Abstract

Understanding carboxylate transport through lipid membranes under physiological conditions is critical in biomedicine and biotechnology, as it allows for the emulation of biological membrane functions and can enhance the absorption of hydrophobic carboxylate-based drugs. However, the structural diversity of carboxylates has made it challenging to study their transport, and the limited available examples do not provide a comprehensive understanding of the role of the organic moiety in this process. Here, we present an in-depth analysis of the diffusion and transport of various aliphatic and aromatic carboxylates into liposomes. We assessed the influence of their size, number of carboxylate groups, and presence of hydroxyl groups. Our findings from fluorescence assays, using lucigenin and HPTS as probes, revealed that most carboxylates can spontaneously diffuse into liposomes in their protonated state, facilitated by the efflux of HNO_3_ when using NaNO_3_ solutions at pH 7. The Cl-ISE assay showed chloride/carboxylate exchange by a synthetic anion transporter. Clear trends were observed when the organic moiety was systematically varied, with a particular enhancement of anion transport by the presence of hydroxyl groups in the aromatic carboxylates. Our findings provide insights into the processes by which carboxylates can enter liposomes, which can contribute to understanding the transport of other biologically relevant organic anions.

## 1. Introduction

The study of ion transport across artificial phospholipid membranes by synthetic transporters has become increasingly significant in recent years as it allows for the emulation of biological membrane functions, including regulation of electrolyte levels and cellular communication [1,2]. However, most studies have focused on the transport of inorganic anions, whereas the transmembrane transport of organic anions has been given less attention. This is surprising, given that organic anions have essential roles in cells [3]. Carboxylates (RCOO^−^) are present in biological molecules such as amino acids, peptides, and proteins [4,5]. Additionally, carboxylates are key molecules in cellular metabolism, biosynthesis, and the release of stored energy through the Krebs cycle. Furthermore, many active pharmaceutical ingredients (APIs) [6] and antibacterial [7] and fungicidal [8] compounds have carboxylate groups in their structures.

Despite the relevance of organic carboxylates in biological systems, only a few papers have reported on the assisted transport of different carboxylates [9,10,11,12,13,14,15] or amino acids [16,17,18] across artificial phospholipid membranes, while acetate is more often included in anion selectivity studies [19,20,21,22,23]. These reports give a first indication that the organic moiety significantly influences the physical–chemical properties of the carboxylates and, consequently, their transport. For instance, the organic group affects the hydrophilicity/hydrophobicity balance of the anion [13] as well as the equilibrium between the anion (RCOO^−^) and its protonated form (RCOOH) [9], which has fewer restrictions to cross the lipid membrane. Additionally, functional groups in the organic moiety increase the complexity of the process as they can interact with the transporter [10], lipid membranes, and aqueous medium. These factors make each carboxylate unique, hampering direct comparison in transmembrane transport experiments. Furthermore, the limited number of carboxylates studied so far limits the understanding of the role of the organic moiety in this process.

**Figure 1:**
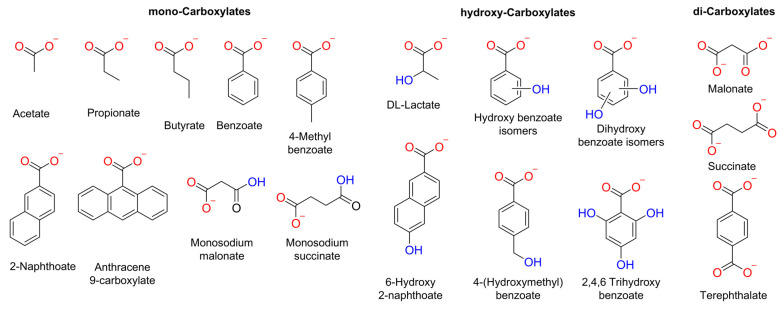
Chemical structure of the carboxylates assessed in this work. These carboxylates were selected considering the following parameters: number of negative charges, −OH groups, and size.

Herein, we conducted a systematic study into the impact of the organic moiety in various carboxylates on their diffusion or transport into liposomes. We examined the size, number of RCOO^−^ groups, and presence of hydroxyl groups in the carboxylate anions (Figure 1). We used various methods to study the spontaneous diffusion of carboxylates, as well as their transport by an *o*-phenylene bisurea anion transporter (**T1**) [24].

## 2. Results

Lucigenin is a well-known fluorescent probe commonly used for anion transport studies [12,13,25]. Before conducting the fluorescence experiments, we evaluated the effect of the various aliphatic and aromatic carboxylates on lucigenin emission. For that, 25 mM of the corresponding carboxylate was added to 0.8 mM lucigenin in 225 mM NaNO_3_, 5 mM HEPES, pH 7. These conditions were selected as they are common buffer solutions for studying anion transport. The carboxylate pulse reduced the lucigenin fluorescence intensity at 505 nm to 31–75% of its initial value, depending on the carboxylate added (Figure S3). Aromatic carboxylates showed more quenching of the lucigenin fluorescence intensity than aliphatic carboxylates. Additionally, dicarboxylates did not show significant differences compared to their monocarboxylate analogues. For comparison, a solution of 25 mM chloride (Cl^−^) was also tested, as it is a well-known lucigenin quencher.

### 2.1. Fluorescence Studies to Monitor the Spontaneous Diffusion of Carboxylates

Our first step was to assess the potential ability of organic carboxylates to diffuse spontaneously into liposomes without the assistance of a molecular transporter, based on previous reports showing indications that carboxylates might cross the lipid membrane without the assistance of a synthetic anion carrier in lucigenin assays [12,13]. To gain insight into this, we selected malonic and succinic acids, as well as their deprotonated forms (mono-deprotonated: monosodium malonate, monosodium succinate; di-protonated: malonate, succinate) based on their high solubility in aqueous solutions (2a). These compounds also allow us to study how each form affects the emission intensity of lucigenin.

**Figure 2:**
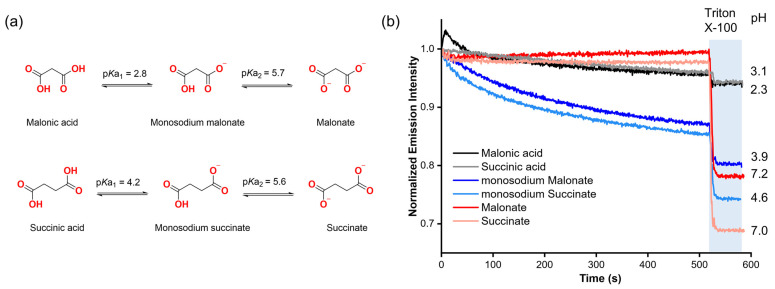
(**a**) Protonation–deprotonation equilibria of malonic and succinic acids. (**b**) Fluorescence changes upon the addition of 25 mM malonic acid, succinic acid, and their deprotonated forms to liposomes with lucigenin encapsulated. The liposome solutions were adjusted to pH 7 with 5 mM HEPES for malonate and succinate, and to pH 5 with 5 mM MES for monosodium malonate, monosodium succinate, malonic acid, and succinic acid experiments.

Malonate and succinate were added to the exterior of liposomes loaded with 0.8 mM lucigenin in 225 mM NaNO_3_, 5 mM HEPES, pH 7, to create a concentration gradient across the liposomal membrane. In both cases, when a pulse of the corresponding dicarboxylate (25 mM) was added in the absence of any transporter, it did not affect the lucigenin emission intensity over time. However, when Triton X-100 (5% *w*/*w* in water) was added, both anions quenched the fluorescence of the lucigenin (Figure 2b).

For monosodium carboxylates, liposomes were loaded with 0.8 mM lucigenin in 225 mM NaNO_3_, 5 mM MES, pH 5. In contrast to the dianions, monosodium malonate and monosodium succinate progressively reduced the lucigenin emission intensity, which supports the previous observations regarding the spontaneous diffusion of carboxylates through lipid membranes. It should be noted that for these anions, the addition of the carboxylate changes the pH value in the liposome solution from 5 to 3.9 and 4.6 for monosodium malonate and monosodium succinate, respectively. These pH values are close to where the maximum fractions for monosodium malonate (96%) and monosodium succinate (85%) are expected based on the calculated speciation graphs (Figure S5).

Finally, malonic and succinic acids were added under the same conditions as monosodium carboxylates. Upon adding malonic or succinic acid to the liposome solution, the pH value dropped from 5 to 2.3 and 3.1, respectively. However, the addition of carboxylic acids and the increased acidity did not have a significant effect on lucigenin emission, even upon lysis. Further experiments using solutions without liposomes showed that neither malonic acid nor succinic acid quenched the fluorescence of lucigenin (Figure S6). These results revealed that RCOO^−^ anions, rather than RCOOH groups, quench the fluorescence of lucigenin. The observed quenching with monosodium malonate and succinate thus indicates that carboxylates end up inside the liposomes.

### 2.2. Mechanism of Carboxylate Diffusion

**Figure 3:**
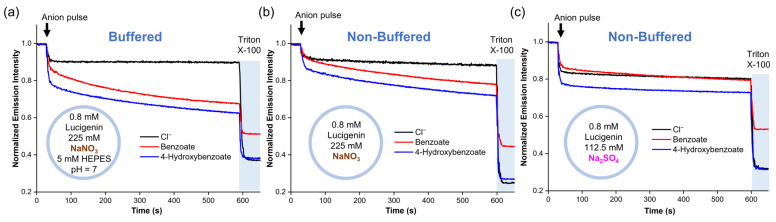
Diffusion of benzoate and 4-hydroxybenzoate into liposomes with lucigenin encapsulated in (**a**) buffered liposome solution in 225 mM NaNO_3_ and 5 mM HEPES (pH 7), (**b**) non-buffered liposome solution in 225 mM NaNO_3_ (pH ≈ 8.1–8.5), and (**c**) non-buffered liposome solution in 112.5 mM Na_2_SO_4_ (pH ≈ 8.2–8.4).

To identify the mechanism by which RCOO^−^ anions diffuse into liposomes without an anion transporter, we conducted experiments using liposomes with different salt solutions. For these experiments, benzoate and 4-hydroxybenzoate were used because they also displayed spontaneous diffusion across the lipid membrane, are present in their anionic form >99.5% at pH 7 (Figure S7), and quench lucigenin more effectively than the aliphatic carboxylates [25]. Accordingly, two liposome solutions with 0.8 mM lucigenin in 225 mM NaNO_3_ were tested. The first solution contained 5 mM HEPES at pH 7, while the second had no buffer. We observed the slow diffusion of carboxylates through the lipid membrane in both samples (Figure 3a,b). This was the case even in the non-buffered liposome solutions, where the global pH became basic after adding the anion pulse (pH 8.1–8.5). It should be noted that pH values < 10 do not significantly affect the fluorescence of lucigenin [25]. Subsequently, NaNO_3_ was replaced by Na_2_SO_4_, and a new liposome solution with 0.8 mM lucigenin in 112.5 mM Na_2_SO_4_ was prepared. Under these conditions, benzoate and 4-hydroxybenzoate addition gave pH values of 8.2–8.4. After a small rapid drop in fluorescence intensity, which was larger for 4-hydroxybenzoate than for benzoate, the intensity remained nearly constant, indicating no diffusion (Figure 3c).

**Figure 4:**
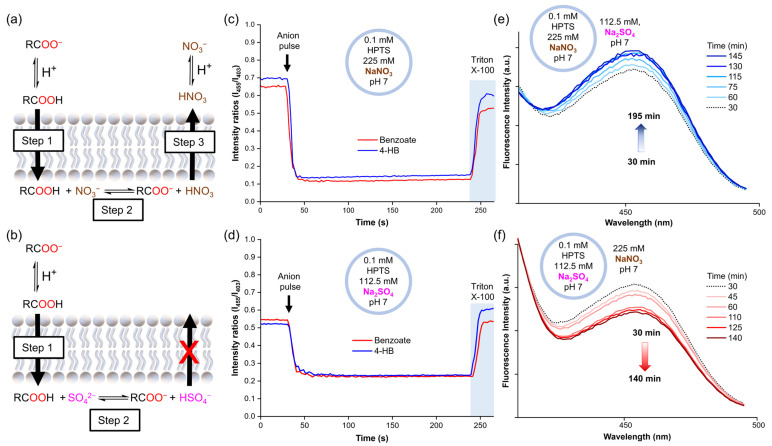
(**a**) Mechanism suggested for carboxylate diffusion in nitrate media, (**b**) mechanism suggested for carboxylate diffusion in sulphate media. The pH inside liposomes after the carboxylate pulse (25 mM sodium benzoate or 4-hydroxybenzoate) as monitored by HPTS fluorescence in (**c**) liposomes with 0.1 mM HPTS in 225 mM NaNO_3_, 5 mM HEPES, pH 7, (**d**) liposomes with 0.1 mM HPTS in 112.5 mM Na_2_SO_4_, 5 mM HEPES, pH 7. Changes in the HPTS excitation band at 455 nm in (**e**) liposomes loaded with 0.1 mM HPTS in 225 mM NaNO_3_, 5 mM HEPES, pH 7, and suspended in 112.5 mM Na_2_SO_4_, 5 mM HEPES, pH 7, (**f**) liposomes loaded with 0.1 mM HPTS in 112.5 mM Na_2_SO_4_, 5 mM HEPES, pH 7, and suspended in 225 mM NaNO_3_, 5 mM HEPES, pH 7.

To obtain further insight into these results, the experiments were repeated with lucigenin replaced by 8-hydroxypyrene-1,3,6-trisulfonic acid trisodium (HPTS). HPTS is a pH-sensitive dye that can be used to monitor the internal pH inside vesicles by monitoring its ratiometric fluorescence intensity (λ_ex_ = 403 and 455 nm, λ_em_ = 510 nm). Accordingly, liposome solutions were prepared with 0.1 mM HPTS in 112.5 mM Na_2_SO_4_, 5 mM HEPES, pH 7, or 225 mM NaNO_3_, 5 mM HEPES, pH 7. Once again, 25 mM benzoate or 4-hydroxybenzoate was added to the liposomes. The HPTS experiments showed that the addition of a carboxylate pulse instantly increased the acidity inside liposomes, after which the pH remained constant over time, both in liposomes suspended in NaNO_3_ and Na_2_SO_4_ (Figure 4c,d). However, this pH decrease was significantly more pronounced for the samples in the NaNO_3_ solution. As no variations in pH were observed outside liposomes upon the addition of the carboxylates, a pH gradient was induced. Thus, these experiments suggest that the carboxylates diffuse across the lipid membrane along with protons, as carboxylic acids.

Combining the results of the lucigenin and HPTS experiments, we hypothesise that carboxylates cross the lipid membrane through a three-step mechanism, driven by the initial concentration gradient of the carboxylates (25 mM exterior and 0 mM interior). Step 1: the formation of small amounts of carboxylic acid in the exterior aqueous solution, which diffuses freely across the lipid membrane. Step 2: the deprotonation of the carboxylic acid inside the liposomes, leading to the formation of free carboxylate and a drop in the pH, which increases the fraction of protonated nitrate (HNO_3_). Step 3: the efflux of HNO_3_ to decrease the pH gradient, followed by its rapid dissociation to recover the NO_3_^−^ anions (Figure 4a). This process allows the slow carboxylic acid diffusion into the liposomes to continue over time, as long as the exterior concentration remains higher than the interior concentration. In contrast, only the first steps can take place in Na_2_SO_4_ solution, as HSO_4_^−^ cannot cross the membrane spontaneously. Consequently, the potential gradient cannot be dissipated, limiting the entry of further RCOOH molecules (Figure 4b).

In order to demonstrate that the diffusion of HNO_3_ across liposomal membranes can occur at neutral pH, we prepared liposomes with internal solutions of 0.1 mM HPTS in 225 mM NaNO_3_, 5 mM HEPES at pH 7, and an external solution of 112.5 mM Na_2_SO_4_, 5 mM HEPES at pH 7. We then monitored the sample by following the excitation band of deprotonated HPTS at 455 nm over time. The 455 nm band displayed a slight increase in intensity, which was attributed to the basification of the liposome interior due to HNO_3_ efflux (Figure 4e and Figure S11a). Conversely, liposomes with an internal solution of 0.1 mM HPTS in 112.5 mM Na_2_SO_4_, 5 mM HEPES at pH 7, and an external solution of 225 mM NaNO_3_, 5 mM HEPES at pH 7 showed acidification of the liposome interior, in this case, attributed to HNO_3_ influx (Figure 4f and Figure S11b). To discard any potential buffer effect of the NaNO_3_ and Na_2_SO_4_ salts themselves, aliquots of 1 M HCl were added to both salt solutions and similar pH values were measured in both solutions (Table S2).

### 2.3. The Role of the Organic Moiety in the Diffusion of Carboxylates

**Figure 5:**
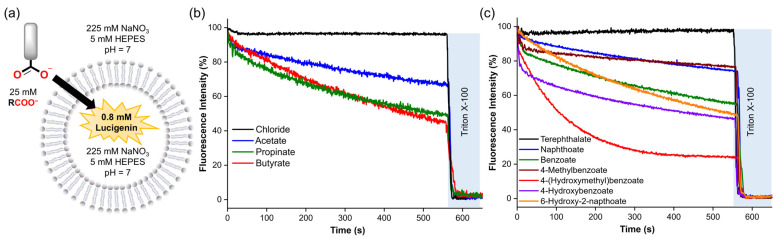
(**a**) Schematic representation of the lucigenin assay, using 0.4 mM lipids, 0.8 mM lucigenin inside, 225 mM NaNO_3_, and 5 mM HEPES (pH 7). (**b**,**c**) Spontaneous diffusion upon addition of 25 mM of the corresponding carboxylate. Fluorescence intensities were normalised from 0–100% since different carboxylates quenched the fluorescence of lucigenin differently.

Subsequently, we assessed the diffusion of various carboxylates into liposomes in NaNO_3_ solutions to study the impact of the organic moiety on this process. Thus, a pulse of the different carboxylates (25 mM) was added to the liposomes loaded with 0.8 mM lucigenin in 225 mM NaNO_3_, 5 mM HEPES at pH 7 (Figure 5a). The anion diffusion was followed by monitoring the decrease in the lucigenin emission over 10 min. Then, liposomes were lysed with Triton X-100 solution.

Consistent with the previous observations, aliphatic and aromatic carboxylates exhibited significant diffusion across the lipid membrane, except for terephthalate, which showed negligible permeability due to its double negative charge [26]. For aliphatic carboxylates, we observed an increase in the diffusion by increasing methylene groups in the organic moiety (butyrate ≥ propionate > acetate) (Figure 5b and Table 1), which is consistent with their calculated LogP values and previous reports concerning the diffusion of carboxylic acids [27,28]. Counterintuitively, aromatic carboxylates with -OH groups showed faster diffusion than their unfunctionalised analogues (Figure 5c). Nevertheless, recent reports describe that some small polar molecules can cross lipid membranes by forming intramolecular H-bonds, which reduces their hydrophilicity [29,30]. Therefore, it is plausible to assume that hydroxyl–carboxylates also might reduce their hydrophilicity by forming H-bond interactions. We note that these experiments could not be performed for all carboxylates, because anions such as citrate, DL-lactate, and anthracene 9-carboxylate resulted in unusual fluorescence behaviour of encapsulated lucigenin (Figure S20), which may be attributed to the reducing effects of citrate and lactate or to potential interactions between lucigenin and the carboxylate inside the liposomes [25].

### 2.4. Assisted Transport of Carboxylates

**Figure 6:**
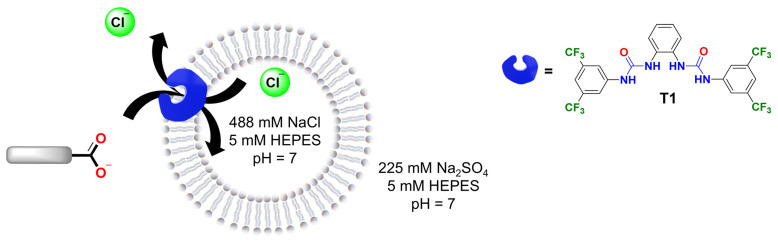
Schematic representation of the Cl^−^/RCOO^−^ exchange by transporter **T1** in liposomes loaded with 488 mM NaCl solution, 5 mM HEPES, pH 7. The carboxylate gradient drives the transport process, while the chloride efflux is monitored with a Cl-ISE.

In the second part of this study, we examined the role of the organic moiety in the assisted transport of carboxylates. To do this, we used a chloride-ion-selective electrode to measure the efflux of Cl^−^ due to electroneutral Cl^−^/RCOO^−^ exchange (Cl-ISE assay, Figure 6) [31]. The Cl-ISE assay was chosen because it allowed us to focus on the transport of carboxylates assisted by a synthetic anion transporter, excluding contributions from the diffusion of the neutral carboxylic acid, which would not result in any Cl^−^ efflux. In addition, the Cl-ISE method enables the direct comparison of various carboxylates, whereas the different carboxylates induce different levels of quenching of lucigenin (Figures S21 and S22).

For these experiments, transporter **T1** (1,1′-(1,2-phenylene)bis(3-(3,5-bis(trifluoromethyl)phenyl)urea)) was selected as it is readily synthesised [24] and similar structures were previously reported to transport carboxylates [9]. Accordingly, liposomes were loaded with 488 mM NaCl, 5 mM HEPES, pH 7 and suspended in a solution of 225 mM Na_2_SO_4_, 5 mM HEPES, pH 7. Then, **T1** in methanol was added to 3 mL of liposome solution with 1 mM lipids in a 1:1000 transporter/lipid ratio and the chloride efflux was monitored for 2 min. After that, the corresponding carboxylate pulse was added (25 mM) and the chloride efflux was measured over 4 min. Finally, liposomes were lysed with Triton X-100 solution to determine the 100% chloride efflux level.

**Figure 7:**
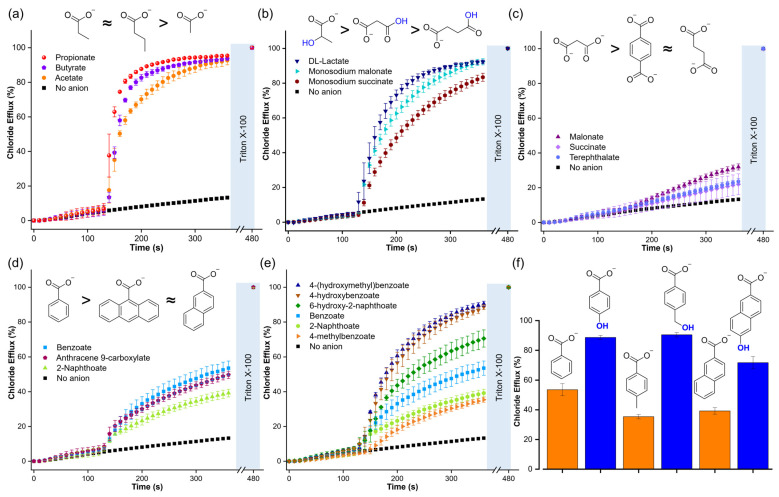
Chloride efflux generated by the Cl^−^/RCOO^−^ exchange upon addition of a pulse of 25 mM of the following compounds: (**a**) aliphatic monocarboxylates, (**b**) aliphatic functionalised carboxylates, (**c**) dicarboxylates, (**d**) aromatic carboxylates with a different number of aromatic rings, (**e**) and (**f**) aromatic carboxylates and comparison with their corresponding analogues hydroxy carboxylates. Liposomes were loaded with 488 mM NaCl, 5 mM HEPES, pH 7, and suspended in a solution of 225 mM Na_2_SO_4_, 5 mM HEPES, pH 7. **T1** was post-inserted in a transporter/lipid ratio of 1:1000. For monosodium malonate and monosodium succinate, liposomes were loaded with 488 mM NaCl, 5 mM MES, pH 5, and suspended in a solution of 225 mM Na_2_SO_4_, 5 mM MES, pH 5.

The Cl-ISE experiments showed that **T1** is highly efficient in transporting aliphatic monocarboxylates (Figure 7a and Table 1), including those with carboxylic acid or hydroxyl functional groups (Figure 7b). These results indicated that a polar functional group did not significantly impact the transport of aliphatic carboxylates. However, dicarboxylates, such as malonate, succinate, and terephthalate, resulted in low chloride efflux, highlighting the challenge of transporting multiply charged anions (Figure 7c). Aromatic carboxylates gave large differences in transport. Interestingly, increasing the size of the aromatic rings from benzoate to 2-naphthoate and anthracene-9-carboxylate did not result in significant variations in the chloride efflux, despite the increase in lipophilicity (Figure 7d and Table 1). However, the addition of aromatic carboxylates with a hydroxyl group resulted in significantly higher Cl^−^/RCOO^−^ exchange rates compared to their unfunctionalised analogues (Figure 7e,f). We note that we observed the same trend for the unassisted diffusion of carboxylates (Figure 5c and Table 1)

### 2.5. Effect of Hydroxyl Groups on the Assisted Transport of Carboxylates

**Figure 8:**
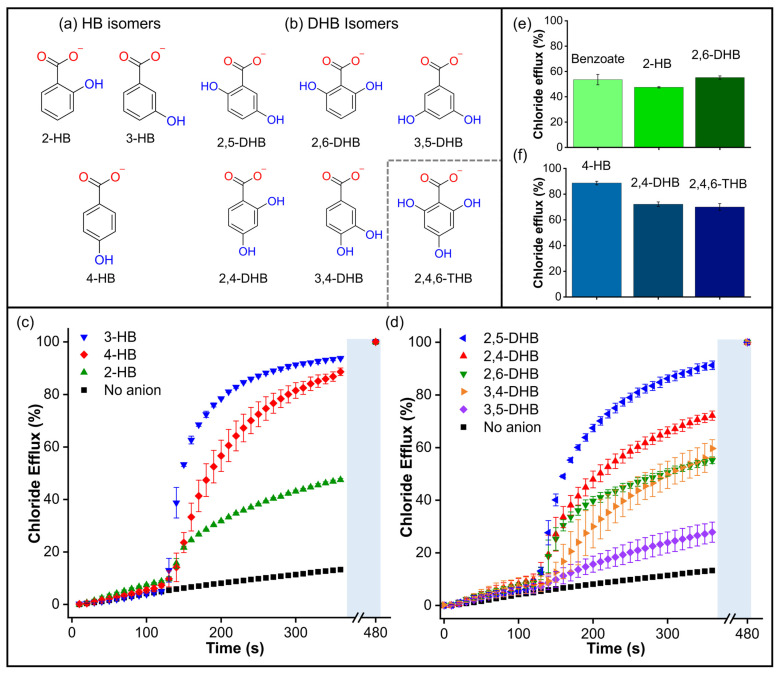
(**a**) Hydroxybenzoate isomers and (**b**) dihydroxybenzoate isomers. Inset: structure of the compound 2,4,6-THB. (**c**) Chloride efflux measured for hydroxybenzoate isomers. (**d**) Chloride efflux measured for dihydroxybenzoate isomers. Comparisons of (**e**) Benzoate, 2-hydroxybenzoate, and 2,6-dihydroxybenzoate; (**f**) 4-Hydroxybenzoate, 2,4-dihydroxybenzoate, and 2,4,6-trihydroxybenzoate. Cl-ISE measurements upon addition of 25 mM of the corresponding carboxylate to liposomes loaded with 488 mM NaCl, 5 mM HEPES, pH 7, and suspended in a solution of 225 mM Na_2_SO_4_, 5 mM HEPES, pH 7. A methanol solution of **T1** was used in all experiments in a 1:1000 transporter/lipid ratio.

In order to further explore the surprising effect of the hydroxyl group on the transport of aromatic carboxylates, we studied isomers of hydroxybenzoate (HB) and dihydroxybenzoate (DHB) (Figure 8a,b). We initially wondered whether the presence of -OH groups could enhance carboxylate transport by stabilizing the negative charge via conjugation. However, the findings for non-conjugated 4-(hydroxymethyl)benzoate suggest that stabilising the negative charge by conjugation is not a crucial factor in this phenomenon. Subsequently, we assessed the transport of 2-hydroxybenzoate (2-HB), 3-hydroxybenzoate (3-HB), and 4-hydroxybenzoate (4-HB), which have different pK_a_ values associated with their charge delocalisation (Table S1). Therefore, if the transport was linked to the charge delocalisation, it was expected to follow the trend 2-HB (pK_a_ = 2.8) > 3-HB (pK_a_ = 3.8) > 4-HB (pK_a_ = 4.4). However, the Cl-ISE results showed better transport for 3-HB and 4-HB, while 2-HB gave similar results to benzoate (pk_a_ = 4.08) (Figure 8c), indicating that the transport trends are independent of the pK_a_ values.

Additionally, we evaluated the effect of the distribution and orientation of H-bonds in the transport of DHB isomers, including 2,5-dihydroxybenzoate (2,5-DHB), 2,4-dihydroxybenzoate (2,4-DHB), 2,6-dihydrozybenzoate (2,6-DHB), 3,4-dihydrozybenzoate (3,4-DHB), and 3,5-dihydroxybenzoate (3,5-DHB) (Figure 8d). We found that additional -OH groups did not necessarily hinder the transport process. In fact, the addition of 2,5-DHB and 2,4-DHB resulted in remarkable transport activity, comparable to that observed with 3-HB and 4-HB. On the other hand, 2,6-DHB and 3,4-DHB were transported at rates similar to benzoate. Finally, 3,5-DHB was transported very slowly, similar to the dicarboxylates. The comparison between benzoate/2-HB/2,6-DHB and 3-HB/2,5-DHB shows that -OH groups in the ortho position do not significantly change the transport rates (Figure 8e and Figure S27). To confirm this observation, we tested the tris-substituted 2,4,6-trihydroxybenzoate (2,4,6-THB). Remarkably, 2,4,6-THB was transported at comparable rates to the 2,4-DHB and 4-HB analogues (Figure 8f). These results reveal that the distribution of the -OH groups is more decisive than the number of -OH groups in the transport of small aromatic carboxylates. However, it is important to note that a higher number of -OH groups in carboxylates could increase their polarity and, therefore, hinder their transport, as observed in multi-substituted aliphatic carboxylates such as D-glucuronate and gluconate (Figure S25) [32].

**Figure 9:**
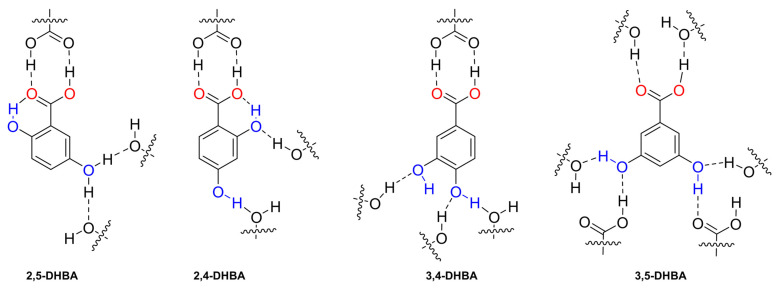
Fragment of the crystalline arrangement of the DHBA isomers and their H-bond inter- and intramolecular interactions indicated with dashed lines. For the 2,5-DHBA isomer, the hydroxyl in the meta position participated in two H-bond interactions with a 50% probability.

We hypothesised that the differences in the transport of DHB isomers were caused by the formation of inter- and intramolecular interactions that may occur between the anion and the surrounding media, lipids, and transporter. To shed some light on this, we analysed the H-bonds present in the crystalline arrangement of 2,5-dihydroxybenzoic acid (2,5-DHBA) [33], 2,4-dihydroxybenzoic acid (2,4-DHBA) [34] 3,4-dihydroxybenzoic acid (3,4-DHBA) [35], and 3,5-dihydroxybenzoic acid (3,5-DHBA) [32] (Figure 9 and Figure S28). The crystal structure of 2,6-dihydroxybenzoic acid (2,6-DHBA) has not been reported. We compared the number of inter- and intramolecular H-bond interactions in each molecule and found that 2,5-DHBA and 2,4-DHBA have a crystalline arrangement where the ortho-hydroxyl participates in a strong intramolecular interaction with the RCOO^−^ group and interacts less with neighbouring molecules. In contrast, 3,4-DHBA and 3,5-DHBA have hydroxyl groups in a position that facilitates the formation of three and four hydrogen bonds, respectively. As a result, we hypothesise that −OH groups in DHB isomers could form similar inter- and intramolecular interactions to their DHBA analogues. Consequently, the larger number of H-bonds observed in 3,4-DHB and 3,5-DHB could disfavour the transport of these carboxylates.

**Figure 10:**
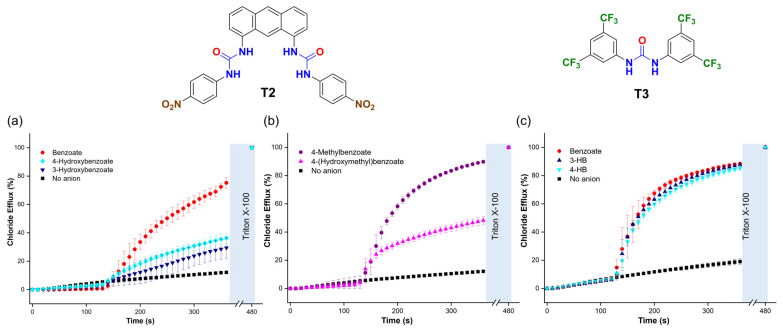
(**a**,**b**) Chloride efflux is promoted upon the addition of 25 mM of the corresponding carboxylate, using **T2** (1:25,000 transporter/lipid ratio) and (**c**) **T3** (1:100 transporter/lipid ratio). Liposomes were loaded with 488 mM NaCl (5 mM HEPES, pH 7) and suspended in a solution of 225 mM Na_2_SO_4_ (5 mM HEPES, pH 7).

To determine if the trends in the transport activity observed for benzoate, 3-hydroxybenzoate (3-HB), and 4-hydroxybenzoate (4-HB) persisted with different transporters, we tested two additional molecules: 1,1′-(anthracene-1,8-diyl)bis(3-(4-nitrophenyl)urea) (**T2**) [36] and 1,3-bis(3,5-bis(trifluoromethyl)phenyl)urea (**T3**) [37]. These transporters were chosen for their distinct organic scaffolds and coordination environments compared to **T1**. We found that **T2**, used in a 1:25,000 transporter/lipid molar ratio, outperformed **T1**. Interestingly, **T2** showed faster transport of benzoate than 4-HB and 3-HB (Figure 10a) and of 4-methylbenzoate than 4-(hydroxymethyl)benzoate (Figure 10b). These trends contrasted with those observed with **T1**, which transported carboxylates with hydroxyl groups at higher rates. On the other hand, **T3**, used at a 1:100 ratio, exhibited lower transport activity overall and showed no significant difference in the transport of benzoate, 3-HB, and 4-HB (Figure 10c). These findings suggest that the interaction between hydroxyl carboxylates and molecular anion transporters is critical for transport efficiency and that the spatial arrangement of the hydrogen bond donors in the anion binding site of the transporters plays an important role in the trends described above. This encourages further investigations into the interaction of other organic anions with transporters to optimise the transport by these compounds.

## 3. Materials and Methods

The diffusion and transport of carboxylates into liposomes were studied by fluorescence spectroscopy using lucigenin and HPTS as probes and by a chloride-ion-selective electrode (Cl-ISE). All the experiments were carried out using 200 nm liposomes prepared from a mixture of 1-palmitoyl-2-oleoyl-sn-glycero-3-phosphocholine (POPC) and cholesterol (ratio = 7:3) following standard methods (see Supporting Information for detailed procedures). The total lipid concentrations used for fluorescence and Cl-ISE experiments were 0.4 mM and 1 mM, respectively.

For the lucigenin assay, the lipid films were hydrated with a solution of 0.8 mM lucigenin (10,10′-dimethyl-9,9′-biacridinium nitrate) and 225 mM NaNO_3_, 5 mM HEPES (2-[4-(2-hydroxyethyl)piperazin-1-yl]ethane-1-sulfonic acid), pH 7. In the HPTS assay, lipid films were hydrated with 0.1 mM HPTS (8-hydroxypyrene-1,3,6-trisulfonic acid trisodium salt) in 225 mM NaNO_3_, 5 mM HEPES, pH 7 or 0.1 mM HPTS in 112.5 mM Na_2_SO_4_, 5 mM HEPES. For the Cl-ISE assay, lipid films were hydrated with a solution of 488 mM NaCl, 5 mM HEPES, pH 7, while an external solution of 225 mM Na_2_SO_4_, 5 mM HEPES, pH 7, was used. For additional details see the Supporting Information.

Sodium carboxylate salts were prepared by reacting the corresponding carboxylic acid (1 equivalent) and sodium hydroxide (1 equivalent for monocarboxylates and 2 equivalents for dicarboxylates) in milli-Q water to get a 1 M concentration (Figures S1 and S2), while sodium acetate was acquired commercially. The concentrations of sodium naphthoate, sodium 6-hydroxy-2-naphthoate, sodium terephthalate, sodium 2,4-dihydroxybenzoate, and sodium 2,4,6-trihydroxybenzoate were reduced to 0.5 M due to their limited solubility in water. It should be noted that carboxylates were prepared in milli-Q water instead of a buffer solution because many of the carboxylates used in this study showed higher solubility in pure water. However, pH measurements were taken before and after each experiment to ensure that the buffer solutions used in each method prevented significant changes in the pH upon the addition of the carboxylate solutions (Table S1). In each experiment, 75 μL of 1 M (or 150 μL of 0.5 M) carboxylate solution was added to 3 mL of liposome to create a gradient of 25 mM.

## 4. Conclusions

We have performed and presented a systematic study to improve our understanding of the spontaneous diffusion and anion transport of organic carboxylates. Using a combination of fluorescence assays and ion-selective electrode experiments in the presence and absence of transporters, we could clearly distinguish both processes.

Our findings reveal that carboxylic acids can diffuse freely into liposomes suspended in NaNO_3_ solutions at pH 7. This is particularly significant as NaNO_3_ is commonly used to study carboxylate transport in large unilamellar vesicles. This highlights the significance of the effect of the medium during transport experiments, particularly in cellular studies.

Furthermore, we found that -OH groups significantly impact carboxylate diffusion and transport, providing new opportunities to explore other functional groups in organic anions and assess their influence in transport experiments. Our findings shed light on how carboxylates are internalised into liposomes and could contribute to the development of carboxylate-based drugs and our understanding of the transmembrane transport of other biologically relevant organic anions. These studies will provide further insights into the diffusion and transport of carboxylates, with potential applications for synthetic transporters in treating channelopathies and improving drug delivery systems.

## Figures and Tables

**Table 1:** Calculated LogP values, relative spontaneous diffusion measured in the lucigenin assay after 570 s (%), and chloride efflux measured with an ISE after 240 s (%) for the carboxylates assessed in this study.

Sample	cLogP ^a^	Diffusion (%) ^b^	Cl^−^ Efflux (%) ^c^
no anion	n/a **^d^**	n/a	13
Aliphatic carboxylates			
Acetate	−3.8	34	92
Propionate	−3.0	50	95
Butyrate	−2.6	55	94
DL-lactate	−4.0	n/a	92
Monosodium malonate	−3.9	65	92
Monosodium succinate	−3.9	55	83
Malonate	−7.4	> 1	32
Succinate	−7.5	5	22
Aromatic carboxylates			
Benzoate	−1.9	45	54
4-methylbenzoate	−1.4	23	35
2-Naphthoate	−0.9	25	39
Anthracene 9-carboxylate	0.1	n/a	50
Terephthalate	−5.8	1	24
Carboxylates with a hydroxyl group			
4-(hydroxymethyl)benzoate	−2.7	76	90
6-hydroxy 2-naphthoate	−1.2	50	72
Hydroxybenzoate isomers (HB)			
2-HB	−1.5	35	47
3-HB	−2.2	35	94
4-HB	−2.2	53	89
Dihydroxybenzoate isomers (DHB)			
2,6-DHB	−1.2	42	55
2,5-DHB	−1.9	40	91
2,4-DHB	−1.9	n/a	72
3,4-DHB	−2.5	42	60
3,5-DHB	−2.5	n/a	28
Trihydroxybenzoate (THB)			
2,4,6-THB	−1.5	52	70

^**a**^ Calculated using MarvinSketch 19.25. ^**b**^ diffusion (%) = 100 × ((I_0_ − I_anion_)/(I_0_ − l_ysis_)). ^**c**^ Chloride efflux (%) = ([Cl^−^]_anion_/[Cl^−^]_lysis_) × 100. ^**d**^ n/a = not applicable.

## Data Availability

The original contributions presented in the study are included in the article and Supplementary Material, further inquiries can be directed to the corresponding author.

## Supplementary Materials

The following supporting information can be downloaded at: https://www.mdpi.com/article/10.3390/molecules29215124/s1, Figures S1 and S2: Chemical structures of carboxylates; Figures S4 and S24: DLS data; Figures S6 and S8: Speciation diagrams; Figures S3 and S7: Fluorescence spectra of lucigenin with different carboxylates; Figures S5 and S9–S23: Fluorescence transport experiments; Figures S25–S28 and S30–S32: Cl-ISE experiments; Figure S29: X-ray structure of DHBA isomers; Table S1: Dissociation constants of carboxylic acids [38,39]; Table S2: pH changes generated by adding aliquots of HCl to NaNO_3_ or Na_2_SO_4_ with 5 mM HEPES.

## References

[1] Davis J.T.P., Gale A., Quesada R. (2020). Advances in Anion Transport and Supramolecular Medicinal Chemistry. Chem. Soc. Rev..

[2] Yang J., Yu G., Sessler J.L., Shin I., Gale P.A., Huang F. (2021). Artificial Transmembrane Ion Transporters as Potential Therapeutics. Chem.

[3] Nigam S.K., Bush K.T., Martovetsky G., Ahn S.Y., Liu H.C., Richard E., Bhatnagar V., Wu W. (2015). The Organic Anion Transporter (OAT) Family: A Systems Biology Perspective. Physiol. Rev..

[4] Skonberg C., Olsen J., Grimstrup Madsen K., Hansen S.H., Grillo M.P. (2008). Metabolic Activation of Carboxylic Acids. Expert Opin. Drug Metab. Toxicol..

[5] Halestrap A.P. (2012). The Monocarboxylate Transporter Family-Structure and Functional Characterization. IUBMB Life.

[6] Lamberth C., Dinges J. (2016). Different Roles of Carboxylic Functions in Pharmaceuticals and Agrochemicals. Bioactive Carboxylic Compound Classes.

[7] Zhu W., Zhang Y., Sinko W., Hensler M.E., Olson J., Molohon K.J., Lindert S., Cao R., Li K., Wang K. (2013). Antibacterial Drug Leads Targeting Isoprenoid Biosynthesis. Proc. Natl. Acad. Sci. USA.

[8] Morgunov I.G., Kamzolova S.V., Dedyukhina E.G., Chistyakova T.I., Lunina J.N., Mironov A.A., Stepanova N.N., Shemshura O.N., Vainshtein M.B. (2017). Application of Organic Acids for Plant Protection against Phytopathogens. Appl. Microbiol. Biotechnol..

[9] Moore S.J., Haynes C.J.E., González J., Sutton J.L., Brooks S.J., Light M.E., Herniman J., Langley G.J., Soto-Cerrato V., Pérez-Tomás R. (2013). Chloride, Carboxylate and Carbonate Transport by Ortho-Phenylenediamine- Based Bisureas. Chem. Sci..

[10] Haynes C.J.E., Berry S.N., Garric J., Herniman J., Hiscock J.R., Kirby I.L., Light M.E., Perkes G., Gale P.A. (2013). Small Neutral Molecular Carriers for Selective Carboxylate Transport. Chem. Commun..

[11] Cossu C., Fiore M., Baroni D., Capurro V., Caci E., Garcia-Valverde M., Quesada R., Moran O. (2018). Anion-Transport Mechanism of a Triazole-Bearing Derivative of Prodigiosine: A Candidate for Cystic Fibrosis Therapy. Front. Pharmacol..

[12] Bak K.M., van Kolck B., Maslowska-Jarzyna K., Papadopoulou P., Kros A., Chmielewski M.J. (2020). Oxyanion Transport across Lipid Bilayers: Direct Measurements in Large and Giant Unilamellar Vesicles. Chem. Commun..

[13] Salam R., Chowdhury S.M., Marshall S.R., Gneid H., Busschaert N. (2021). Increasing Membrane Permeability of Carboxylic Acid-Containing Drugs Using Synthetic Transmembrane Anion Transporters. Chem. Commun..

[14] Alonso-Carrillo D., Arias-Betancur A., Carreira-Barral I., Fontova P., Soto-Cerrato V., García-Valverde M., Pérez-Tomás R., Quesada R. (2023). Small Molecule Anion Carriers Facilitate Lactate Transport in Model Liposomes and Cells. iScience.

[15] Arias-Betancur A., Fontova P., Alonso-Carrillo D., Carreira-Barral I., Duis J., García-Valverde M., Soto-Cerrato V., Quesada R., Pérez-Tomás R. (2024). Deregulation of Lactate Permeability Using a Small-Molecule Transporter (Lactrans-1) Disturbs Intracellular PH and Triggers Cancer Cell Death. Biochem. Pharmacol..

[16] Wu X., Gale P.A. (2016). Small-Molecule Uncoupling Protein Mimics: Synthetic Anion Receptors as Fatty Acid-Activated Proton Transporters. J. Am. Chem. Soc..

[17] Martínez-Crespo L., Sun-Wang J.L., Sierra A.F., Aragay G., Errasti-Murugarren E., Bartoccioni P., Palacín M., Ballester P. (2020). Facilitated Diffusion of Proline across Membranes of Liposomes and Living Cells by a Calix[4]Pyrrole Cavitand. Chem.

[18] Maslowska-Jarzyna K., Bąk K.M., Zawada B., Chmielewski M.J. (2022). pH-Dependent Transport of Amino Acids across Lipid Bilayers by Simple Monotopic Anion Carriers. Chem. Sci..

[19] Vargas Jentzsch A., Emery D., Mareda J., Nayak S.K., Metrangolo P., Resnati G., Sakai N., Matile S. (2012). Transmembrane Anion Transport Mediated by Halogen-Bond Donors. Nat. Commun..

[20] Benz S., Macchione M., Verolet Q., Mareda J., Sakai N., Matile S. (2016). Anion Transport with Chalcogen Bonds. J. Am. Chem. Soc..

[21] Shinde S.V., Talukdar P. (2019). Transmembrane H^+^/Cl^−^ Cotransport Activity of Bis(Amido)Imidazole Receptors. Org. Biomol. Chem..

[22] Valkenier H., Akrawi O., Jurček P., Sleziaková K., Lízal T., Bartik K., Šindelář V. (2019). Fluorinated Bambusurils as Highly Effective and Selective Transmembrane Cl^−^/HCO_3_^−^ Antiporters. Chem.

[23] Singh A., Torres-Huerta A., Vanderlinden T., Renier N., Martínez-Crespo L., Tumanov N., Wouters J., Bartik K., Jabin I., Valkenier H. (2022). Calix[6]Arenes with Halogen Bond Donor Groups as Selective and Efficient Anion Transporters. Chem. Commun..

[24] Martínez-Crespo L., Halgreen L., Soares M., Marques I., Félix V., Valkenier H. (2021). Hydrazones in Anion Transporters: The Detrimental Effect of a Second Binding Site. Org. Biomol. Chem..

[25] Chvojka M., Singh A., Cataldo A., Torres-Huerta A., Konopka M., Šindelář V., Valkenier H. (2024). The Lucigenin Assay: Measuring Anion Transport in Lipid Vesicles. Anal. Sens..

[26] Gregory K.P., Elliott G.R., Robertson H., Kumar A., Wanless E.J., Webber G.B., Craig V.S.J., Andersson G.G., Page A.J. (2022). Understanding Specific Ion Effects and the Hofmeister Series. Phys. Chem. Chem. Phys..

[27] Walter A., Gutknecht J. (1984). Monocarboxylic Acid Permeation through Lipid Bilayer Membranes. J. Membr. Biol..

[28] Li S., Hu P.C., Malmstadt N. (2011). Imaging Molecular Transport across Lipid Bilayers. Biophys. J..

[29] Rezai T., Yu B., Millhauser G.L., Jacobson M.P., Lokey R.S. (2006). Testing the Conformational Hypothesis of Passive Membrane Permeability Using Synthetic Cyclic Peptide Diastereomers. J. Am. Chem. Soc..

[30] Alex A., Millan D.S., Perez M., Wakenhut F., Whitlock G.A. (2011). Intramolecular Hydrogen Bonding to Improve Membrane Permeability and Absorption in beyond Rule of Five Chemical Space. MedChemComm.

[31] Jowett L.A., Gale P.A. (2018). Supramolecular methods: The chloride/nitrate transmembrane exchange assay. Supramol. Chem..

[32] Jowett L.A., Howe E.N.W., Wu X., Busschaert N., Gale P.A. (2018). New Insights into the Anion Transport Selectivity and Mechanism of Tren-Based Tris-(Thio)Ureas. Chem.-Eur. J..

[33] Cohen D.E., Benedict J.B., Morlan B., Chiu D.T., Kahr B. (2007). Dyeing Polymorphs: The MALDI Host 2,5-Dihydroxybenzoic Acid. Cryst. Growth Des..

[34] Parkin A., Adam M., Cooper R.I., Middlemiss D.S., Wilson C.C. (2007). Structure and Hydrogen Bonding in 2,4-Dihydroxybenzoic Acid at 90, 100, 110 and 150 K; a Theoretical and Single-Crystal X-Ray Diffraction Study. Acta Crystallogr. Sect. B Struct. Sci..

[35] Sarma B., Sanphui P., Nangia A. (2010). Polymorphism in Isomeric Dihydroxybenzoic Acids. Cryst. Growth Des..

[36] Dias C.M., Valkenier H., Davis A.P. (2018). Anthracene Bisureas as Powerful and Accessible Anion Carriers. Chem.-Eur. J..

[37] Busschaert N., Kirby I.L., Young S., Coles S.J., Horton P.N., Light M.E., Gale P.A. (2012). Squaramides as Potent Transmembrane Anion Transporters. Angew. Chem. Int. Ed..

[38] Haynes W.M. (2016). CRC Handbook of Chemistry and Physics.

[39] Armarego W.L.F., Chai C.L.L. (2009). Purification of Laboratory Chemicals.

