# Supplementary material for: The Role of the Organic Moiety in the Diffusion and Transport of Carboxylates into Liposomes
Source: Molecules. 2024 Oct 30;29(21):5124. doi: 10.3390/molecules29215124 (PMC11547798; doi:10.3390/molecules29215124)
Supplement: Supplementary file 1 [file molecules-29-05124-s001.zip › molecules-3260430-supplementary.pdf]

Supporting Information

The Role of the Organic Moiety in the Diffusion and Transport of Carboxylates into Liposomes

Aaron Torres-Huerta, Hennie Valkenier*

Université libre de Bruxelles (ULB), Engineering of Molecular NanoSystems, Ecole polytechnique de Bruxelles, Avenue F. Roosevelt 50, CP165/64, B-1050 Brussels, Belgium.

hennie.valkenier@ulb.be

Table of Contents

1. General experimental information ... 2

2. Preparation of sodium carboxylate salts ... 2

3. Fluorescence quenching of lucigenin by carboxylates ... 5

4. General procedure for the preparation of liposomes ... 5

5. Fluorescence studies on liposomes and carboxylates ... 6

5.1 Lucigenin assay ... 6

5.2 Fluorescence quenching of lucigenin by malonic acid, succinic acid, and their deprotonated forms ... 7

5.3 HPTS experiments ... 8

5.4 Spontaneous diffusion of carboxylates ... 12

5.5 Assisted transport of carboxylates using the transporter T1 ... 16

6. Carboxylate transport using a chloride ion-selective electrode (Cl-ISE) ... 17

7. Proton transport by monensin using the Cl-ISE assay ... 20

1. General experimental information

The reagents and solvents used in this study were obtained from Sigma Aldrich, Fluorochem, Alfa Aesar and VWR. Reagents were used without any further purification unless otherwise stated. 1-palmitoyl-2-oleoyl-*sn*-glycero-3-phosphocholine (POPC) and cholesterol were purchased from Sigma Aldrich and Acros, respectively. Chloroform was deacidified to prepare the lipid solutions by passing it through a column containing basic alumina. POPC solutions were stored at -20 °C, while the cholesterol solutions were freshly prepared. All aqueous solutions were prepared using deionised water passed through a Millipore filtration system. Liposomes were used within 4 hours of preparation.

Fluorescence measurements were performed on a FluoroMax-4 (Horiba) spectrofluorometer equipped with a water-thermostatted cell holder with stirring and an injection port.

A Fisherbrand Accumet AB250 chloride selective electrode was used for determining chloride concentration during transport experiments. The chloride electrode was calibrated against solutions of $NaNO_3$ 0.5 M with sodium chloride in concentrations of 1 ppm, 10 ppm, 100 ppm and 1000 ppm, respectively.

Dynamic Light Scattering (DLS) measurements were performed on a Malvern Zetasizer Ultra at 25 ˚C, using disposable cuvettes to assess the average size and size distribution. For liposome solutions, a standard refractive index of 1.45 was used.

2. Preparation of sodium carboxylate salts

Sodium carboxylate salts were prepared by reacting the corresponding carboxylic acid (1 equivalent) and sodium hydroxide (1 equivalent for monocarboxylates and 2 equivalents for dicarboxylates) in 1 ml of milli-Q water to get a 1 M concentration (Figure S1 and S2). For sodium naphthoate, sodium 6-hydroxy-2-naphthoate, sodium terephthalate, sodium 2,4-dihydroxybenzoate and sodium 2,4,6-trigydroxybenzoate the concentration was reduced to 0.5 M because of their limited solubility in water. Additionally, pH values were measured for each experiment after adding the corresponding carboxylate in the liposome solution in all experiments (Table S1).

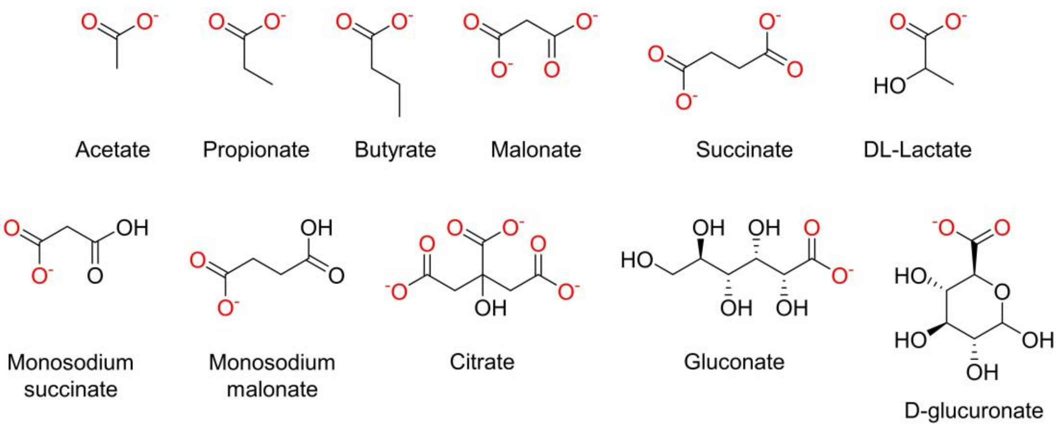

Figure S1: Chemical structure of the aliphatic carboxylates studied in this work.

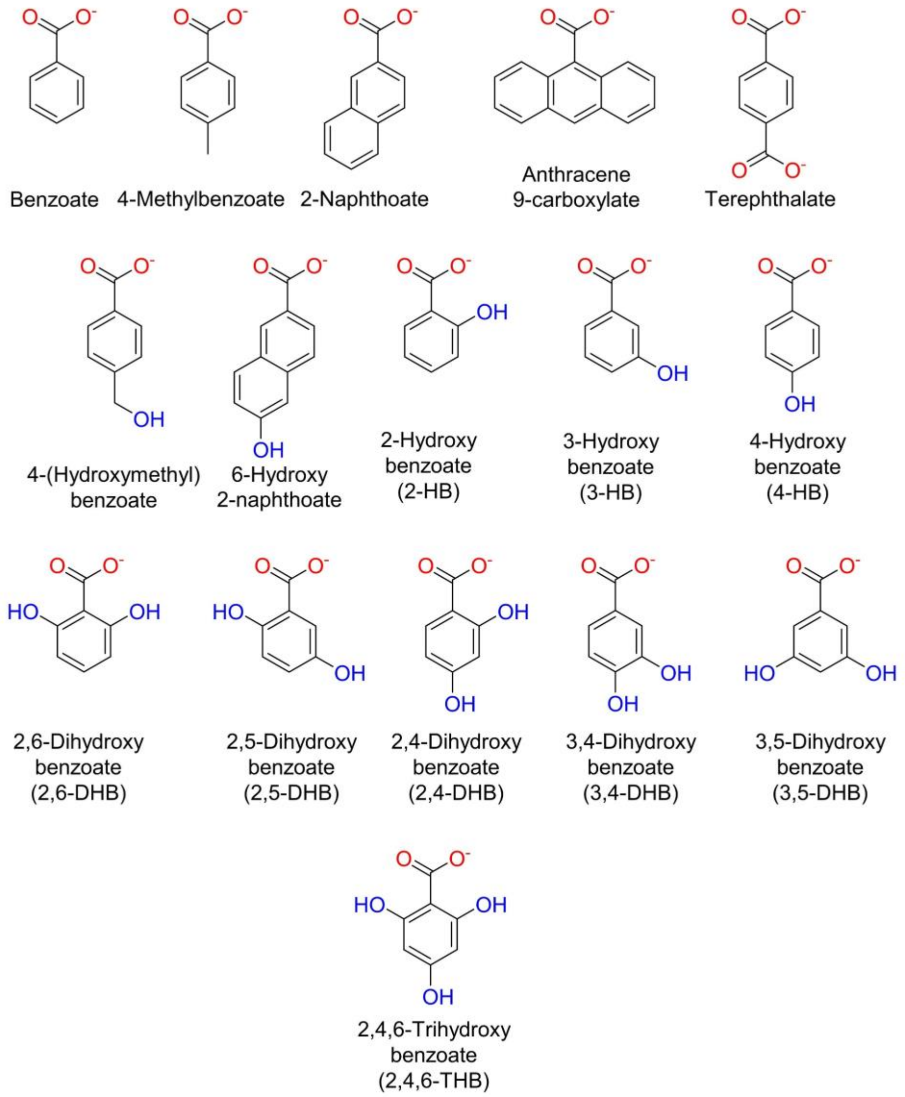

Figure S2: Chemical structure of the aromatic carboxylates studied in this work.

Table S1: Dissociation constants of carboxylic acids studied in this work.

**Carboxylic acid**	pK_a1[1]	pK_a2[1]<br>pk_a3[1]	Experimental pH[4]
**Aliphatic carboxylates**			
Acetic acid	4.5 / 4.7[2]		7.2
Propionic acid	4.7 / 4.9[2]		7.0
Butyric acid	4.9 / 4.8[2]		7.0
DL-lactic acid	3.8 / 3.9[2]		7.0
Malonic acid	2.4 / 2.8[2]	5.9 / 5.7[2]	2.3 / 3.9 / 7.2
Succinic acid	3.5 / 4.2[2]	5.7 / 5.6[2]	3.1 / 4.6 / 7.0
Citric acid	3.0 / 3.3[2]	4.7 / 4.8[2]<br>5.4 / 6.4[2]	7.2
Gluconic acid	3.4		7.1
Glucuronic acid	3.2 / 3.2[3]		7.2
**Aromatic carboxylates**			
Benzoic acid	4.1 / 4.2[2]		7.0
4-Methylbenzoic acid	4.3 / 4.4[2]		7.2
2-Naphthoic acid	4.0 / 4.1[3]		7.2
Anthracene 9-carboxylic acid	3.2 / 3.6[3]		7.3
Terephthalic acid	3.3 / 3.5[2]	4.6 / 4.3[2]	7.4
4-(Hydroxymethyl)benzoic acid	4.1	15.0	7.0
6-Hydroxy 2-naphthoic acid	4.0	9.8	6.8
**Hydroxybenzoate isomers (HB)**			
2-Hydroxybenzoic acid	2.8 / 3.0[2]	13.2 / 13.6[2]	7.2
3-Hydroxybenzoic acid	3.8 / 4.1[2]	9.6 / 9.9[2]	7.3
4-Hydroxybenzoic acid	4.4 / 4.6[2]	9.7 / 9.5[2]	7.0
**Dihydroxybenzoate isomers (DHB)**			
2,6-Dihydroxybenzoic acid	1.6 / 1.1[4]	13.2<br>14.5	7.6
2,5-Dihydroxybenzoic acid	2.5 / 3.0[2]	10.0<br>15.5	6.8
2,4-Dihydroxybenzoic acid	3.1 / 3.1[2]	9.8 / 8.6[2]<br>13.3 / 14.0[2]	6.7
3,4-Dihydroxybenzoic acid	4.2 / 4.5[2]	9.4 / 8.8[2]<br>12.8 / 12.6[2]	6.9
3,5-Dihydroxybenzoic acid	3.6 / 4.0[2]	13.2<br>14.5	7.0
**Trihydroxybenzoate isomer (THB)**			
2,4,6-Trihydroxybenzoic acid	1.9 / 1.5[3]	$pK_a2 = 9.9$<br>$pK_a3 = 13.4$<br>$pK_a4 = 14.7$	7.5

[1] Calculated using MarvinSketch 19.25.
[2] W. M. Haynes, D. R. Lide and T. J. Bruno *CRC handbook of chemistry and physics*, 2016-2017, 97th Edition.
[3] W. L. F. Armarego and C. L. L. Chai *Purification of Laboratory Chemicals*, 2009, 6th Edition.
[4] Experimental pH measured after the addition of the corresponding carboxylate in each sample.

3. Fluorescence quenching of lucigenin by carboxylates

A stock solution of 0.8 mM lucigenin in 225 mM $NaNO_3$ and 5 mM HEPES at pH 7 was prepared for these experiments. Then, 3 µL of the stock solution was diluted to 3 ml with the salted solution. The fluorescence spectrum (excitation at 430 nm, 2 nm slits, 0.1 s integration time, 25 °C) was measured before and after the addition of the corresponding 1 M carboxylate solution (75 µL) or a 1 M NaCl solution (75 µL). The fluorescence spectra were collected from 450 to 650 nm, and the intensity was plotted in arbitrary units (Figure S3). The experiments were performed in triplicate.

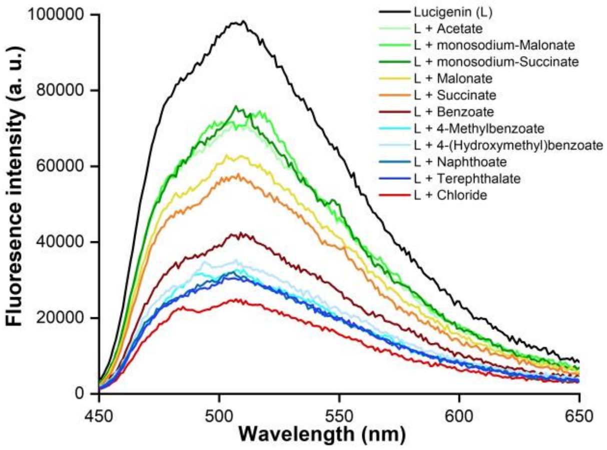

Figure S3: Fluorescence spectra of lucigenin in the presence of various carboxylates (25 mM) in 225 mM $NaNO_3$ and 5 mM HEPES at pH 7. Results are the average of 3 repetitions.

4. General procedure for the preparation of liposomes

Liposomes were prepared from a mixture of 1-palmitoyl-2-oleoyl-sn-glycero-3-phosphocholine (POPC) Phospholipid and cholesterol (ratio 7:3) in deacidified chloroform in a 5 mL round-bottom flask. The volumes were calculated to obtain a final concentration of 0.4 mM (POPC + cholesterol). Chloroform was evaporated under nitrogen, and the resulting lipid film was dried under vacuum overnight. The lipid film was then hydrated with 500 µL of an aqueous solution of 10,10'-dimethyl-9,9'-biacridinium nitrate (lucigenin, 0.8 mM) in a solution of $NaNO_3$ (225 mM) and HEPES (5 mM) at pH 7. The mixture was sonicated for 1 min and stirred for 1 h at room temperature. The heterogeneous multilamellar vesicles were broken down into unilamellar vesicles by 10 freeze-thawing cycles, diluted to 1 mL with the salted solution, and extruded 29 times through polycarbonate membranes (200 nm pore size) at room temperature. To remove external lucigenin, the liposome solution was passed through size exclusion columns (Sephadex G-25), eluted with the salted solution. Finally, the resulting vesicles were diluted to obtain a final lipid concentration of 0.4 mM (calculated from the initial quantities of lipids). Additionally, DLS measurements were conducted in each liposome bath to verify

the appropriate size distribution. Figure S22 displays a representative graph of the liposome solution used in the fluorescence studies.

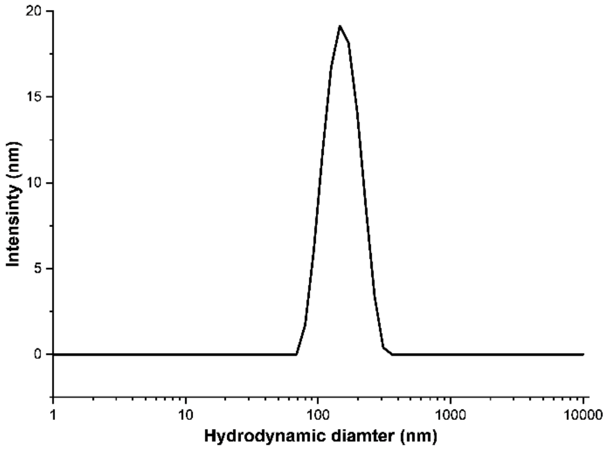

Figure S4. Typical size distribution of liposomes used in the fluorescence studies.

5. Fluorescence studies on liposomes and carboxylates

5.1 Lucigenin assay

Fluorescence measurements were performed on 3 mL of the final vesicle solution in a quartz cuvette with a stir bar, and the temperature was allowed to stabilize at 25 ˚C for 3 min inside the sample compartment of a Fluoromax-4 spectrometer. The temperature of the cuvette holder was maintained at 25 °C using a water bath. The fluorescence intensity of lucigenin was recorded using an excitation wavelength of 430 nm and an emission wavelength of 505 nm. Accordingly, 75 µL of the corresponding 1 M carboxylate solution was added to 3 mL of liposome solution 30 seconds after starting the measurement (to get a concentration of 25 mM), and the fluorescence was monitored for an additional 10 minutes. Finally, liposomes were lysed with 60 µL of Triton X-100 (5 wt.%) in water. Each experiment was repeated at least three times, and the fluorescence data were averaged after removing the initial drop (due to the quenching of the remaining external fluorophore). The normalization process was done using the maximum intensity measured or as per cent. In order to ensure the quality of the liposomes, a pulse of 75 µL of 1M NaCl was used before each experiment.

5.2 Fluorescence quenching of lucigenin by malonic acid, succinic acid, and their deprotonated forms

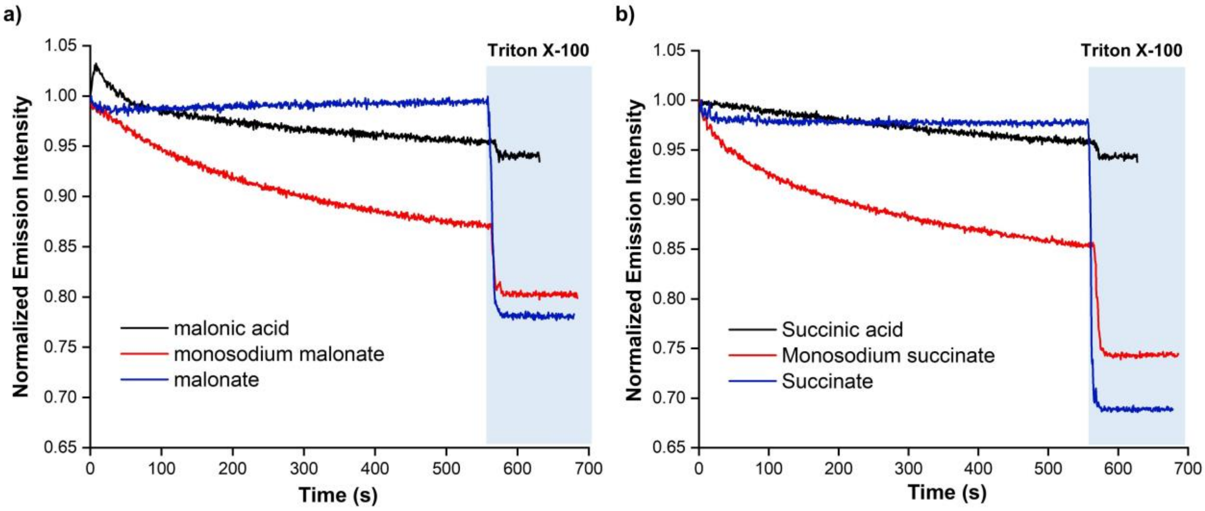

Figure S5: Fluorescence emission changes by adding malonic acid, succinic acid, and their unprotonated forms in the lucigenin experiments. For malonate and succinate, liposomes liposome solutions with 0.8 mM lucigenin were suspended in a solution of 225 mM $NaNO_3$ and 5 mM HEPES, pH 7. For the acids and monoprotonated forms, liposome solutions with 0.8 mM lucigenin were suspended in a solution of 225 mM $NaNO_3$ and 5 mM MES, pH 5.

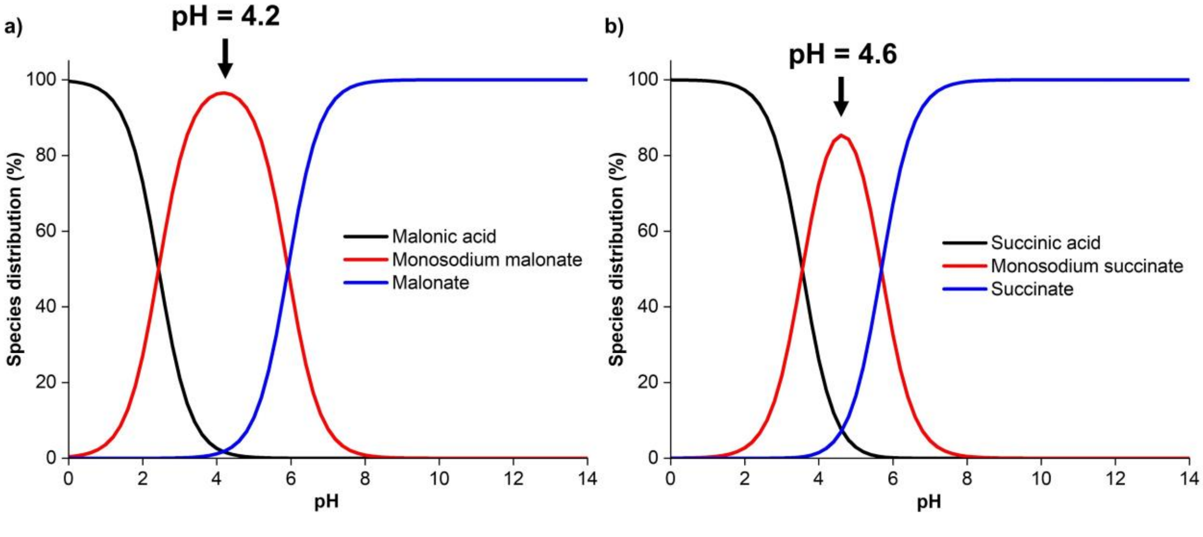

Figure S6: Speciation diagram of malonic and succinic acids. Data points were calculated using the MarvinSketch 19.25 software.

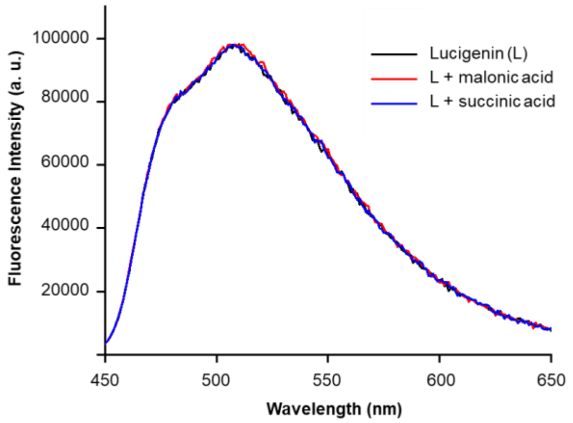

Figure S7: Fluorescence quenching of lucigenin by the presence of succinic acid (pH 3.1), monosodium succinate (pH 4.6) and succinate (pH 7.0).

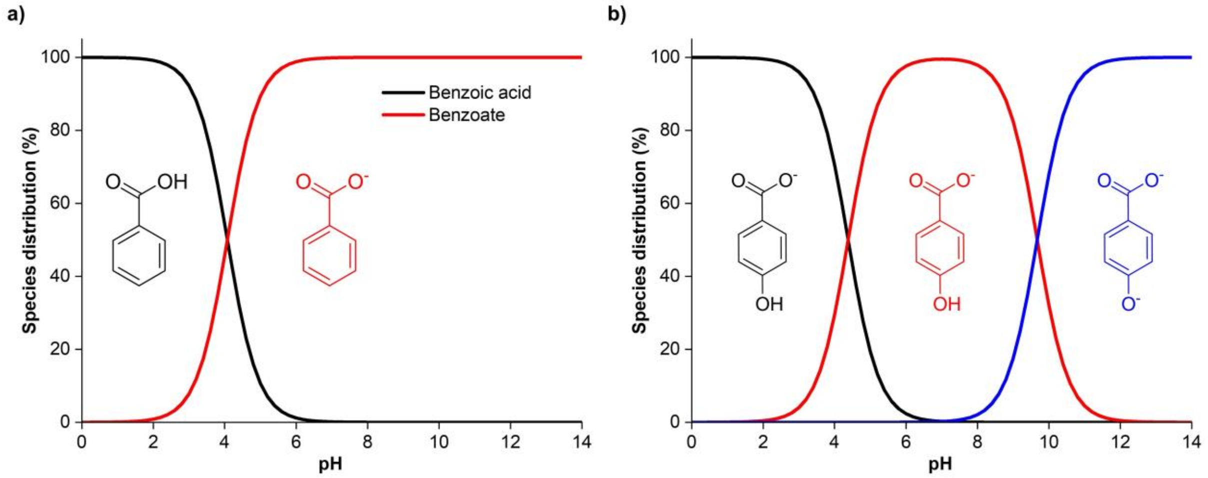

Figure S8: Speciation diagram of benzoic acid and 4-hydroxybenzoic acid. Data points were calculated using the MarvinSketch 19.25 software.

5.3 HPTS experiments

Liposome solutions for the experiments were prepared using the same protocol as previously described. However, lipid film was hydrated with 0.1 mM HPTS in 225 mM $NaNO_3$, 5 mM HEPES, pH 7. or 0.1 mM HPTS in 112.5 mM Na_2SO_4, 5 mM HEPES, pH 7.0. The fluorescence measurements were taken by placing 3 mL of liposomes solution in a quartz cuvette with a small stir bar. The temperature was allowed to stabilize at 25 ˚C for 3 min inside the sample compartment of a Fluoromax-4 spectrometer. The fluorescence intensities (excitation at 403 and 455 nm, emission at 511 nm) were measured over time at 25 ˚C. After 30 seconds, 75 µL of the corresponding 1M carboxylate solution was added to 3 mL of liposome solution. The intensities changes were monitored for 200 seconds, and then liposomes were lysed by adding 60 µL of Triton X-100 (5% w/w in water). The data points were plotted using the fluorescence intensity with excitation at 455 nm (corresponding to the HPTS deprotonated form) divided by the fluorescence intensity with excitation at 403 nm (corresponding

to the HPTS protonated form). The ratios of intensity values were normalized from 0 to 1. All experiments were made in triplicate.

For these experiments, benzoate and 4-hydroxybenzoate were selected. The pH gradient, generated for adding the corresponding carboxylate (25 mM), was monitored over time (Figure S8). The experiments were replicated using a carboxylate pulse of 3 mM to prevent any unexpected effects (Figure S9). However, the pH changes were still notably greater for liposomes suspended in 225 mM $NaNO_3$, 5 mM HEPES, pH 7.

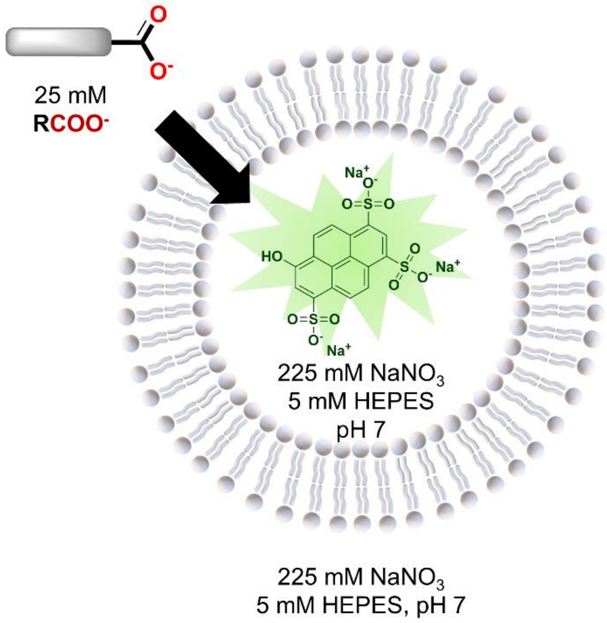

Figure S9: Conditions used for the HPTS assay.

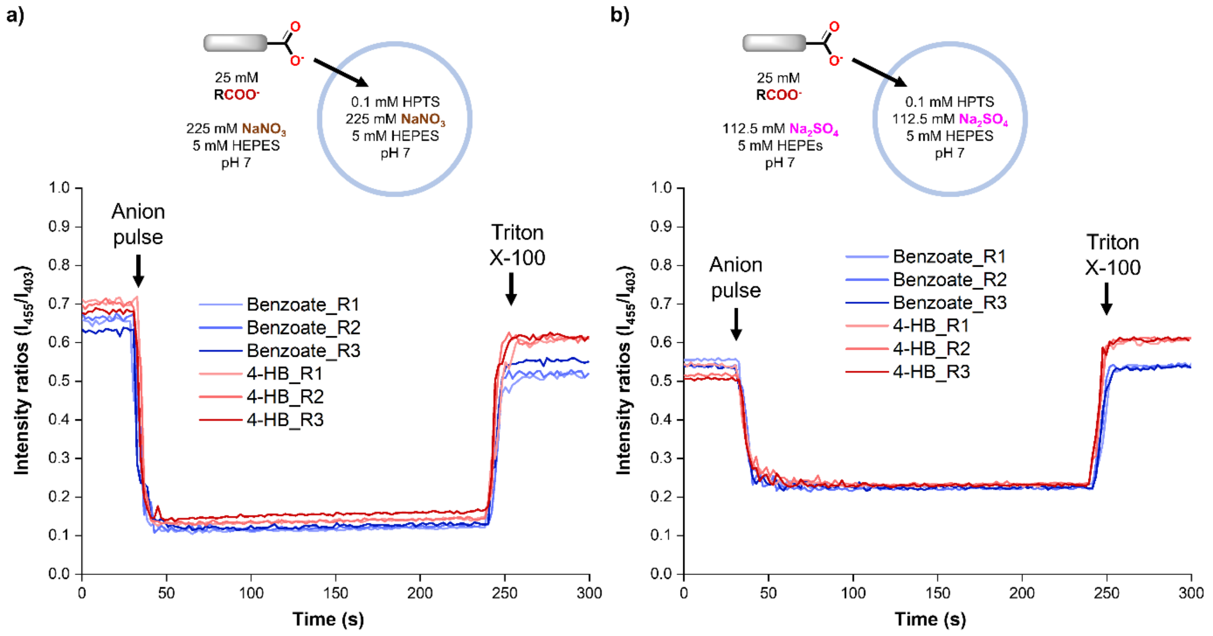

Figure S10: HPTS assay for benzoate and 4-hydroxybenzoate using: a) Liposome prepared with 0.1 mM HPTS in 225 mM $NaNO_3$, 5 mM HEPES, pH 7.0; b) Liposome with 0.1 mM HPTS in 112.5 mM Na_2SO_4, 5 mM HEPES, pH 7.0.

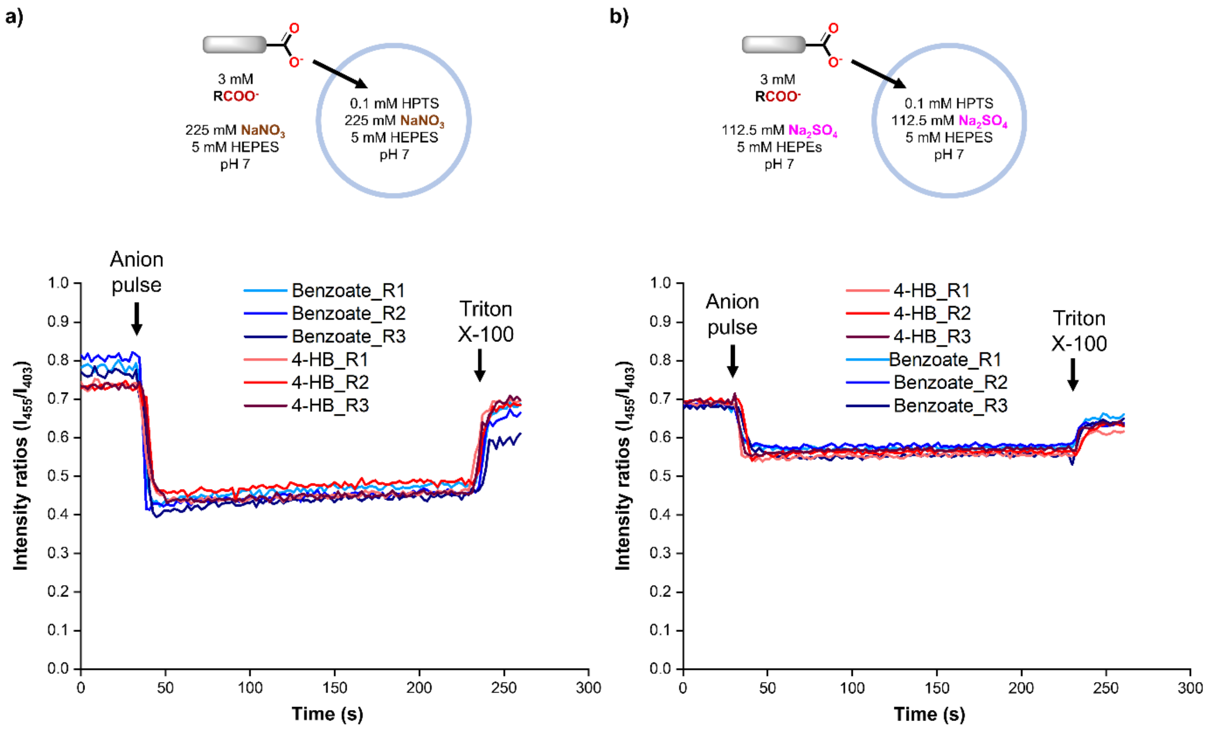

Figure S11: HPTS assay for benzoate and 4-hydroxybenzoate using a anion pulse of 3 mM: a) Liposome prepared with 0.1 mM HPTS in 225 mM $NaNO_3$, 5 mM HEPES, pH 7; b) Liposome with 0.1 mM HPTS in 112.5 mM Na_2SO_4, 5 mM HEPES, pH 7.

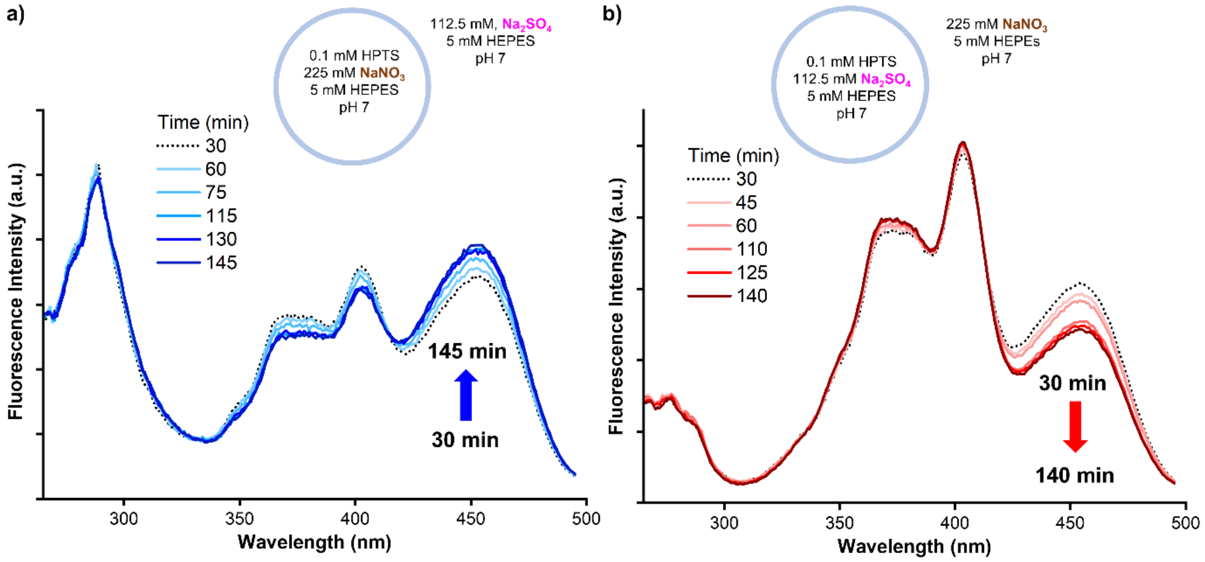

Figure S12: Emission spectra of HPTS using: a) Liposomes loaded with 0.1 mM HPTS in 225 mM $NaNO_3$, 5 mM HEPES, pH 7, and suspended in 112.5 mM Na_2SO_4, 5 mM HEPES, pH 7; b) Liposomes loaded with 0.1 mM HPTS in 112.5 mM Na_2SO_4, 5 mM HEPES, pH 7 and suspended in 225 mM $NaNO_3$, 5 mM HEPES, pH 7.

Table S2: pH changes generated by adding aliquots of 250 mM HCl to 3mL of 225 mM NaNO3, 5 mM HEPES and 112.5 mM Na2SO4, 5 mM HEPES.

	3 mL of 225 mM $NaNO_3$, 5 mM HEPES	3 mL of 112.5 mM Na_2SO_4, 5 mM HEPES
HCl (250 mM)	pH	pH
0 µL	7.0	7.0
5 µL	6.5	6.4
10 µL	6.3	5.9
15 µL	5.5	4.7
20 µL	4.2	4.1

5.4 Spontaneous diffusion of carboxylates

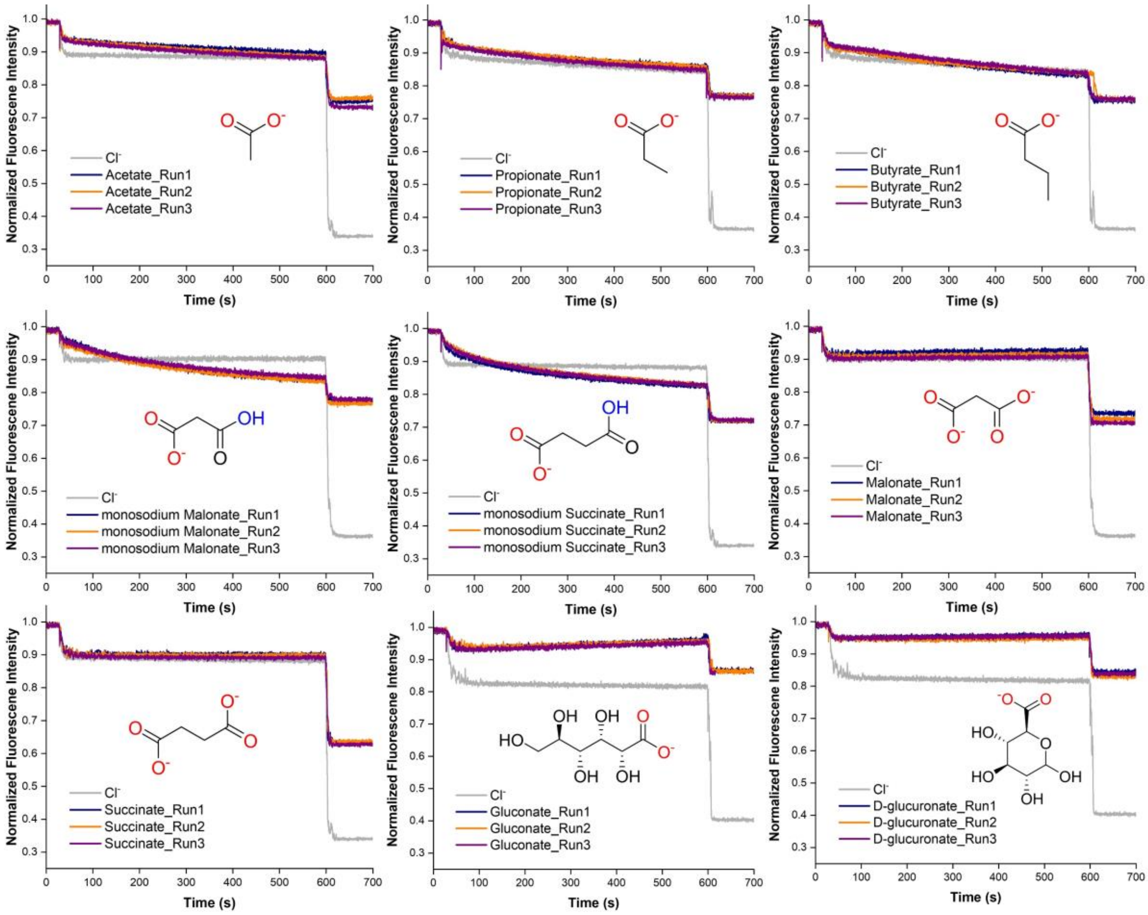

Figure S13: Plots of the entire process for monitoring the spontaneous diffusion of aliphatic carboxylates. Fluorescence intensity has been normalized over to the maximum intensity value.

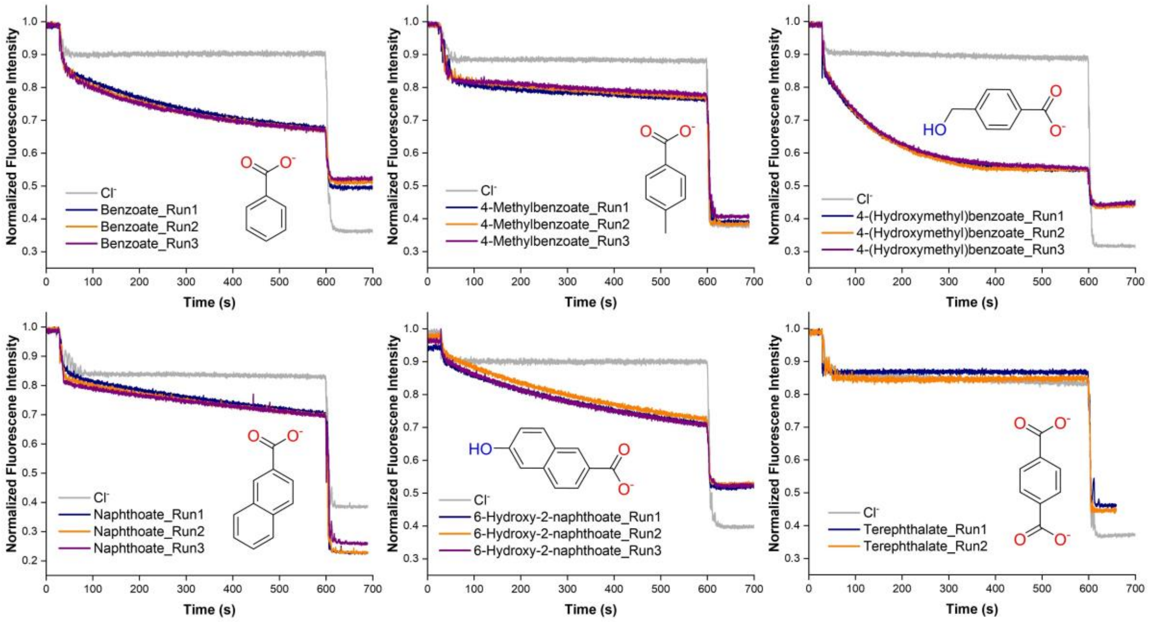

Figure S14: Plots of the entire process for monitoring the spontaneous diffusion of aromatic carboxylates. Fluorescence intensity has been normalized over to the maximum intensity value.

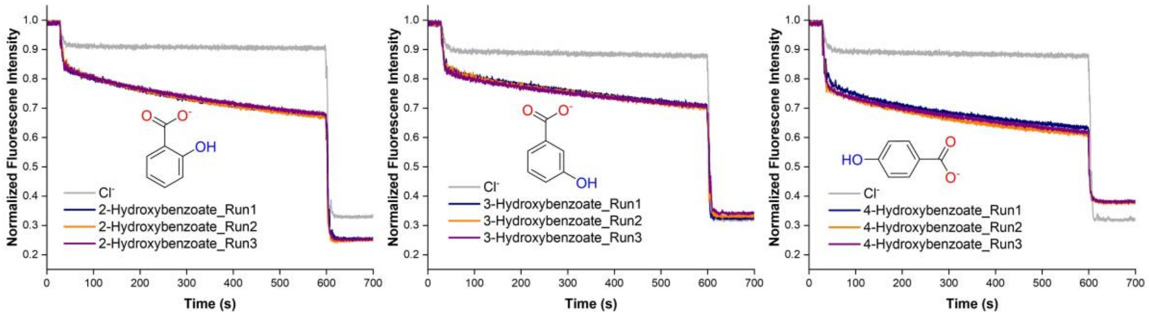

Figure S15: Plots of the entire process for monitoring the spontaneous diffusion of hydroxybenzoate isomers (HB). Fluorescence intensity has been normalized over to the maximum intensity value.

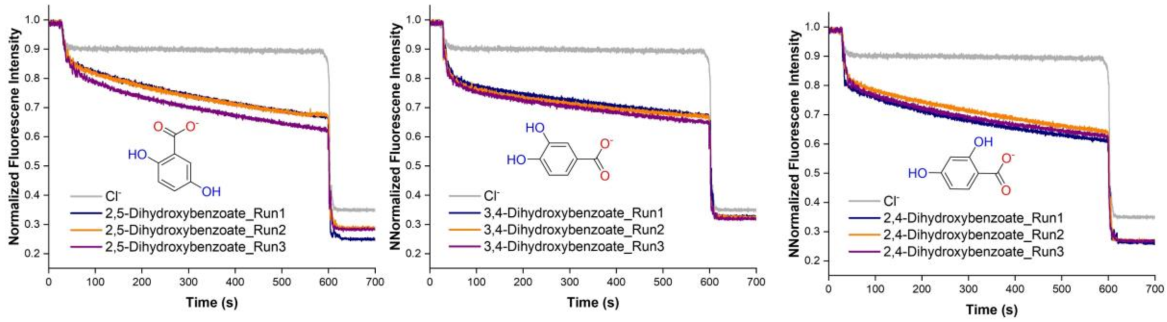

Figure S16: Plots of the entire process for monitoring the spontaneous diffusion of dihydroxybenzoate isomers (DHB). Fluorescence intensity has been normalized over to the maximum intensity value.

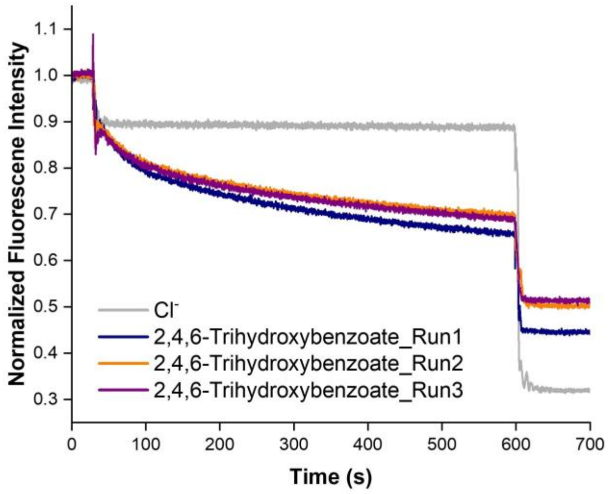

Figure S17: Plots of the entire process for monitoring the spontaneous diffusion of the 2,4,6-trihydroxybenzoate. Fluorescence intensity has been normalized over to the maximum intensity value.

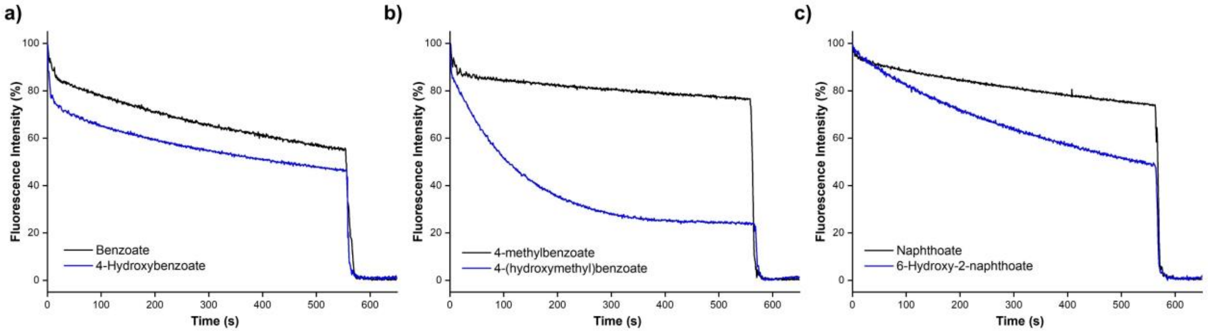

Figure S18: Spontaneous diffusion of the compounds: a) Benzoate and 4-hydroxybenzoate; b) 4-methylbenzoate and 4-(hydroxymethyl)benzoate. The fluorescence data was averaged and normalized to 100% for comparison purposes. The initial intensity drop was removed for clarity.

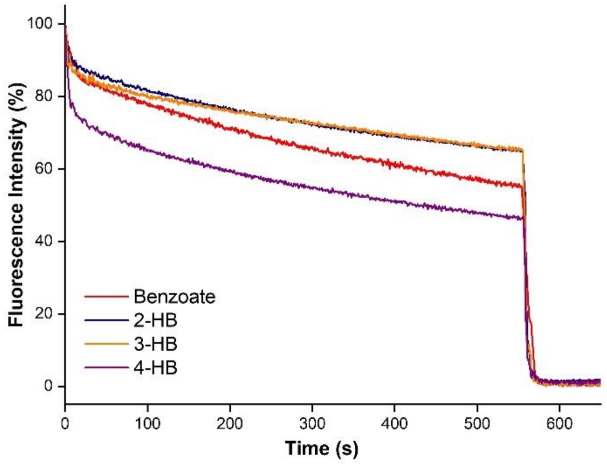

Figure S19: Spontaneous diffusion of benzoate and the hydroxybenzoate isomers (HB). The fluorescence data was averaged and normalized to 100% for comparison purposes. The initial intensity drop was removed for clarity.

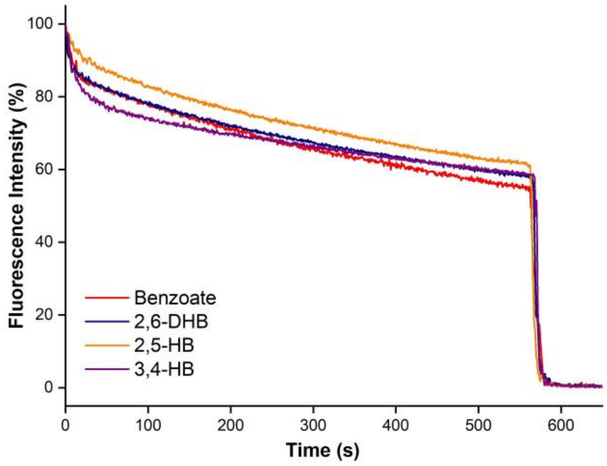

Figure S20: Spontaneous diffusion of benzoate and the dihydroxybenzoate isomers (DHB). The fluorescence data was averaged and normalized to 100% for comparison purposes. The initial intensity drop was removed for clarity.

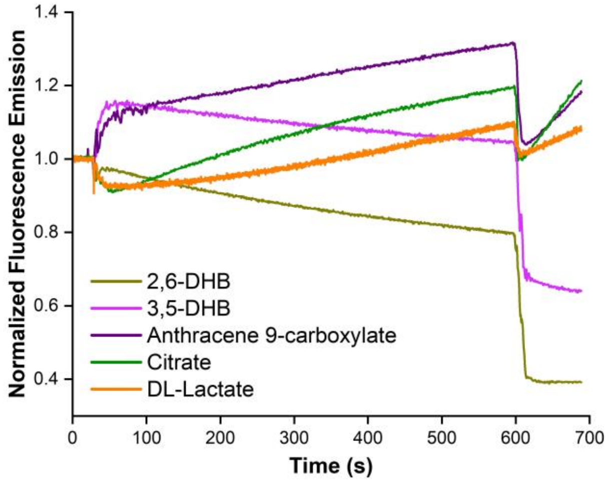

Figure S21: Unusual fluorescence spectra of the carboxylates: 2,6-dihydroxybenzoate, 3,5-dihydroxybenzoate, anthracene 9-carboxylate, citrate, and DL-lactate. The normalization process was done using the intensity value at the beginning of each measurement.

5.5 Assisted transport of carboxylates using the transporter T1

The liposome solutions for the experiments were prepared using the same protocol and conditions as previously described. However, the transporter **T1** in methanol was added in a transporter lipid ratio of 1:1000 before the temperature stabilization process. The data points were then normalized using the maximum intensity measured. Accordingly, benzoate, 4-methylbenzoate and 2-naphtholate were assessed as those carboxylates showed lower spontaneous diffusion and significantly quenched the lucigenin emission intensity. Additionally, malonate, succinate and terephthalate were tested but they showed negligible transport.

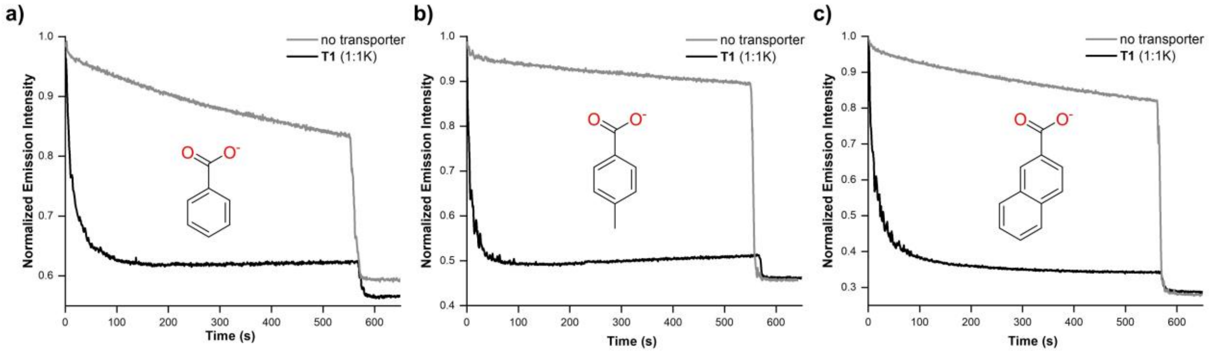

Figure S22: Transport of various aromatic carboxylates monitored by using the lucigenin assay. a) Benzoate; b) 4-Methylbenzoate; c) Naphthoate. Liposome in 225 mM $NaNO_3$ and 5 mM HEPES at pH 7. Transporter **T1** was post-inserted in a transporter lipid ratio of 1:1000. Experiments were made in triplicate, and data points were averaged and normalized to the maximum intensity. The initial intensity drop was removed for clarity.

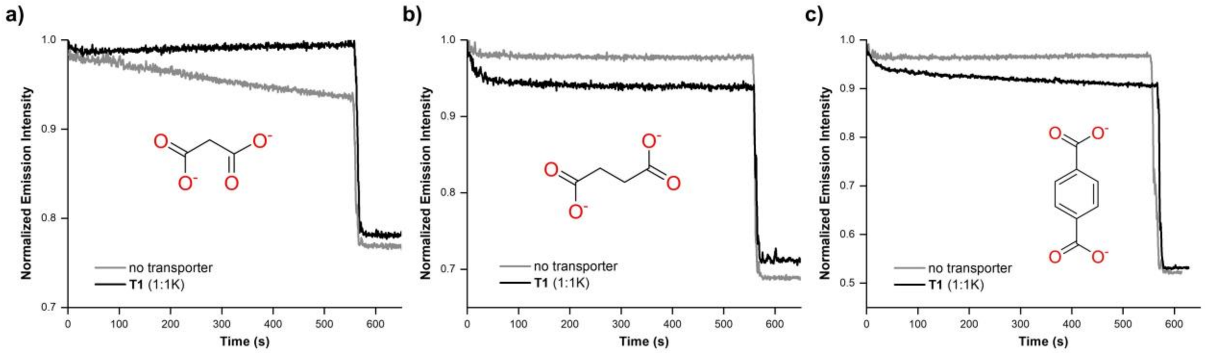

Figure S23: Transport of various dicarboxylates monitored by using the lucigenin assay. a) Malonate; b) Succinate; c) Terephthalate. Liposome in 225 mM $NaNO_3$ and 5 mM HEPES at pH 7. The transporter **T1** was post-inserted in a transporter lipid ratio of 1:1000. Experiments were made in triplicate, and data points were averaged and normalized to the maximum intensity. The initial intensity drop was removed for clarity.

6. Carboxylate transport using a chloride ion-selective electrode (Cl-ISE)

Liposomes were prepared using the same protocol and conditions as previously described. However, lipids were hydrated with a solution of 488 mM NaCl, 5 mM HEPES, pH 7. To remove the excess of NaCl outside liposomes, liposome solution was purified three times by dialysis using a Biotech CE Tubing dialysis membrane (MWCO 20kD) and an external solution of 225 mM Na_2SO_4, 5 mM HEPES, pH 7. The resulting solution was diluted with the external solution until it reached a lipid concentration of 1 mM. Chloride efflux was monitored using a Fisherbrand Accument AB250 chloride ion-selective electrode, which was calibrated before each experiment. The Cl-ISE assay used 3 mL of the liposome solution. The transporter **T1** in methanol and a transporter lipid ratio of 1:1000 was added to the liposome solution, and the corresponding carboxylate was added 120 seconds later. The chloride efflux was monitored for 4 min, and then liposomes were lysed with 100 µL of Triton X-100 (5% w/w in water) to determine 100% chloride efflux. All experiments were made in triplicate, and a blank experiment without transporters was made to ensure the quality of liposomes before each experiment. Additionally, DLS measurements were made in each liposome bath to verify the proper size distribution (Figure S22).

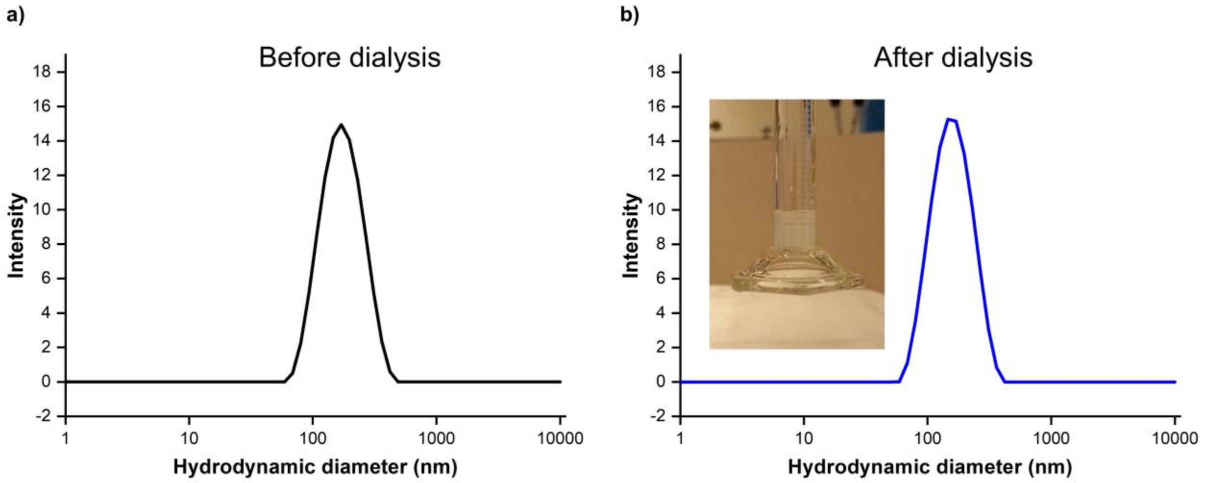

Figure S24: Hydrodynamic diameter of the liposome solution before and after to performed the dialysis process. Inserted, photographic of the liposome solution.

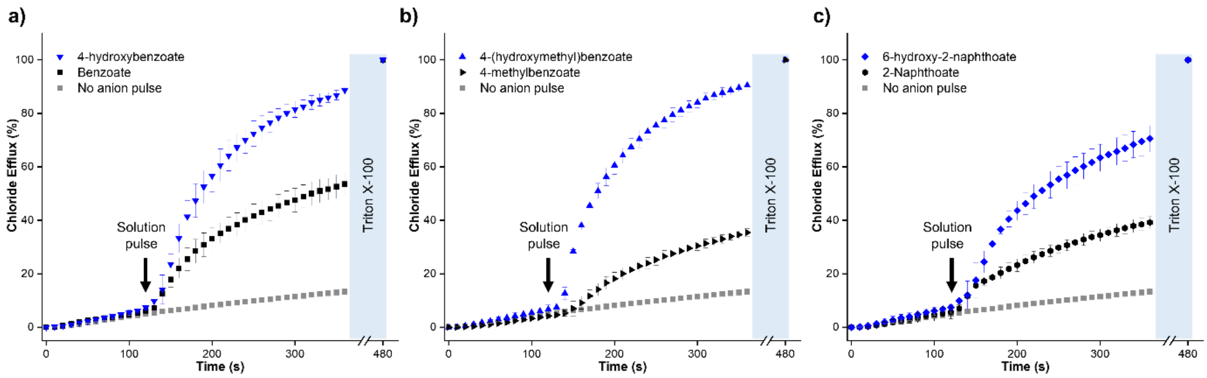

Figure S25: Chloride efflux generated by the $Cl^-/RCOO^-$ exchange for the carboxylate groups: a) Benzoate/4-hydroxybenzoate; b) 4-Methylbenzoate/4-(Hydroxymethyl)benzoate; c) 2-Naphthoate/6-hydroxy-2-naphthoate. Liposomes were loaded with 488 mM NaCl, 5 mM HEPES, pH 7 and suspended in a solution of 225 mM Na_2SO_4, 5 mM HEPES, pH 7. Transporter **T1** was post-inserted in a transporter lipid ratio of 1:1000.

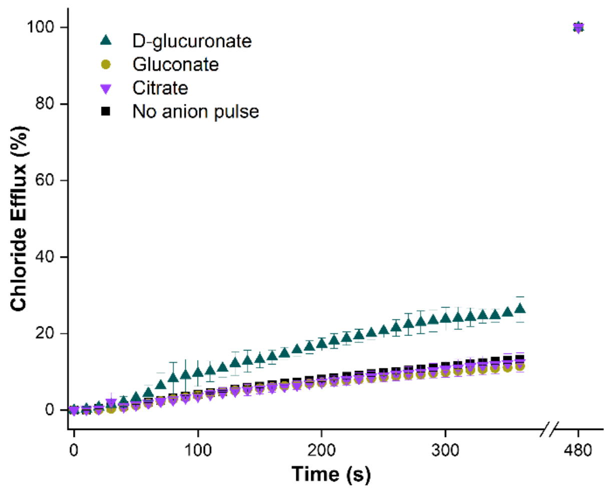

Figure S26: Chloride efflux generated by the $Cl^-/RCOO^-$ exchange for the carboxylate: citrate, gluconate and D-glucuronate. Liposomes were loaded with 488 mM NaCl, 5 mM HEPES, pH 7 and suspended in a solution of 225 mM Na_2SO_4, 5 mM HEPES, pH 7. Transporter **T1** was post-inserted in a transporter lipid ratio of 1:1000.

We also tested malonic and succinic acids and their deprotonated forms without the assistance of **T1,** using the Cl-ICE assay. However, we observed negligible chloride efflux (≤3%) after adding the corresponding pulse (Figure S26). The results show that liposomes remain stable under acidic conditions and indicate that carboxylates do not create defects in the lipid membrane that could result in chloride leaks.

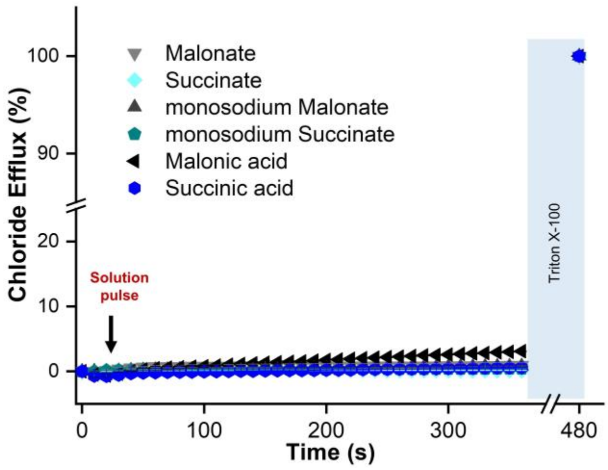

Figure S27: Chloride efflux generated by the $Cl^-/RCOO^-$ exchange for the malonic and succinic acids and their unprotonated forms. Liposomes were loaded with 488 mM NaCl, 5 mM HEPES, pH 7 and suspended in a solution of 225 mM Na_2SO_4, 5 mM HEPES, pH 7.

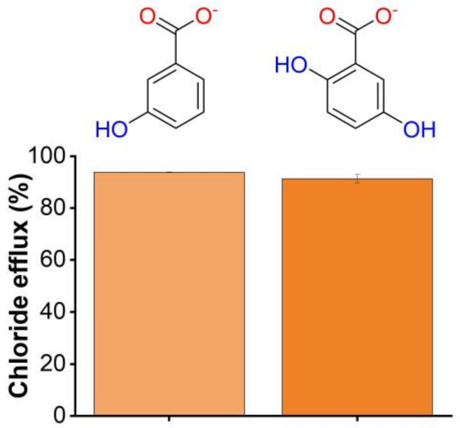

Figure S28: Chloride efflux comparison for; 3-hydroxybenzoate and 2,5-dihydroxybenzoate.

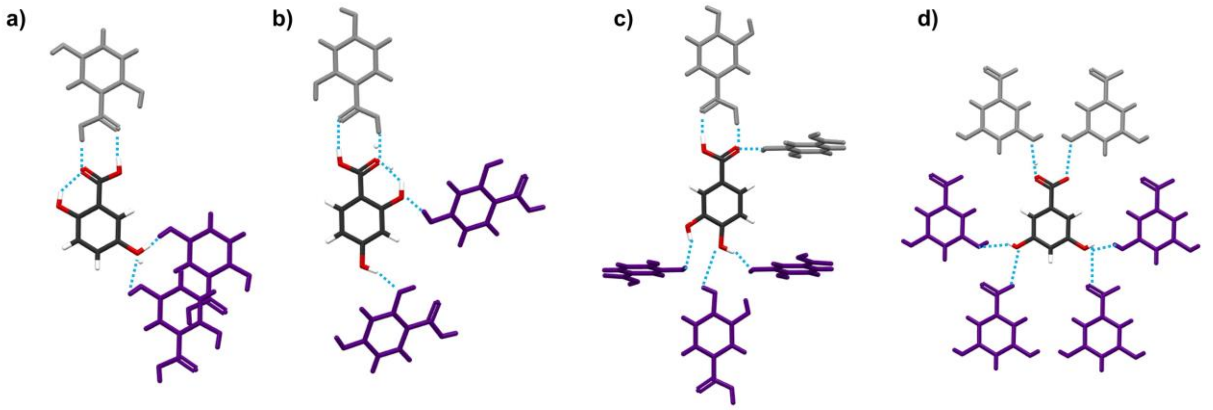

Figure S29: Fragment of the crystalline arrangement of the DHBA isomers showing the inter and intramolecular H-bonds found for a) 2,5-Dihydroxybenzoic acid (2,5-DHBA); b) 2,4-Dihydroxybenzoic acid (2,4-DHBA); c) 3,4-Dihydroxybenzoic acid (3,4-DHBA); and d) 3,5-Dihydroxybenzoic acid (3,5-DHBA). For the 2,5-DHBA isomer, the hydroxyl group in the meta position participates in two different H-bond interactions with a 50% probability for each one.

7. Transport studies with monensin using the Cl-ISE assay

Considering that the bis-urea transporters are often able to conduct HCl transport, we tested the liposome solutions in the presence of the corresponding transporter (**T1**, **T2** and **T3**) and monensin, which is a proton and cation transporter (Figure S29). Experiments were performed using the same standard conditions described for the Cl-ISE assay, but no carboxylates were added. All transporters showed HCl transport in the presence of monensin (Figure S30).

These findings suggest the possibility of an alternative route for the observed chloride efflux during the carboxylate transport experiments, involving the spontaneous diffusion of the carboxylic acid into the liposomes in combination with the transport of HCl by the transports to prevent the increasing formation of a pH gradient (Figure S31a). However, our experiments showed that the spontaneous diffusion observed for the HB and DHB isomers is similar, especially for the 2-HB and 3-HB isomers (Figure S31c and S31e), whereas significant differences were observed for the carboxylate transport by **T1** in the Cl-ISE assay (Figure 30b and 30d). Furthermore, the carboxylate transport happens faster than the spontaneous diffusion process. Therefore, the spontaneous diffusion of carboxylic acids cannot be the main cause of the observed Cl^- efflux, confirming that carboxylates are indeed transported by **T1**, as indicated schematically in Figure 6.

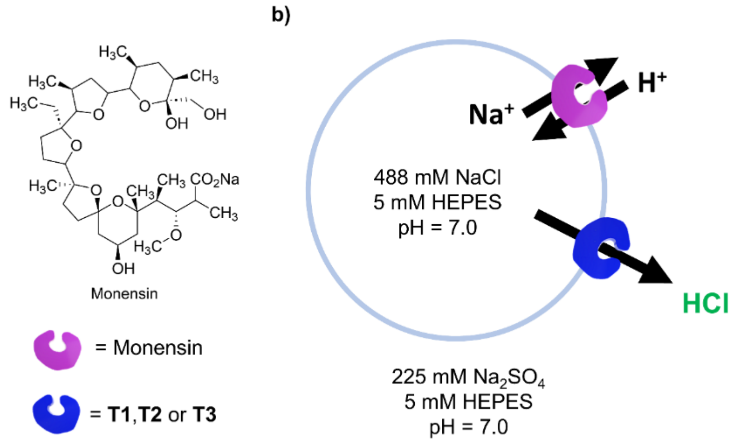

Figure S30: Schematic representation of the experiments performed with monensin and the conditions used. Lipids (1 mM). Internal solution: 488 mM NaCl, 5 mM HEPES, pH = 7.0. Internal solution: 225 mM Na_2SO_4, 5 mM HEPES, pH = 7.0.

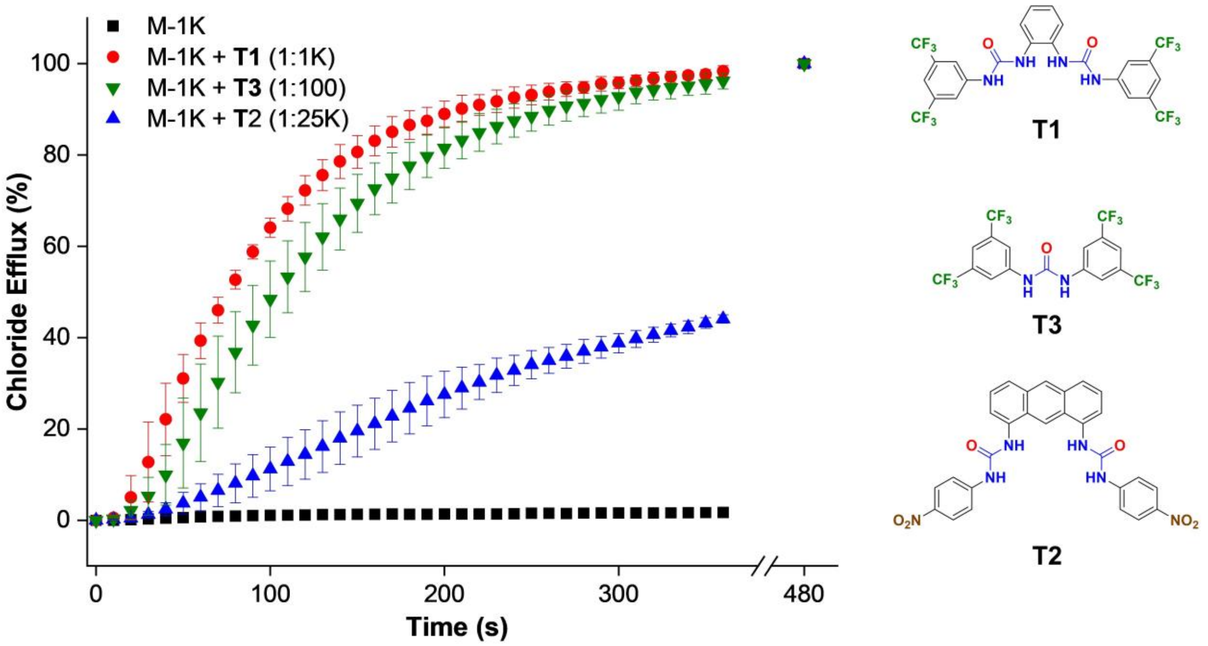

Figure S31: Chloride efflux generated by monensin and the bis-urea transporter. T1, T2 and T3 were post-inserted in a lipid ratio of 1:1000, 1:25000 and 1:100, respectively. Internal solution: 488 mM NaCl, 5 mM HEPES, pH = 7.0. Internal solution: 225 mM Na_2SO_4, 5 mM HEPES, pH = 7.0.

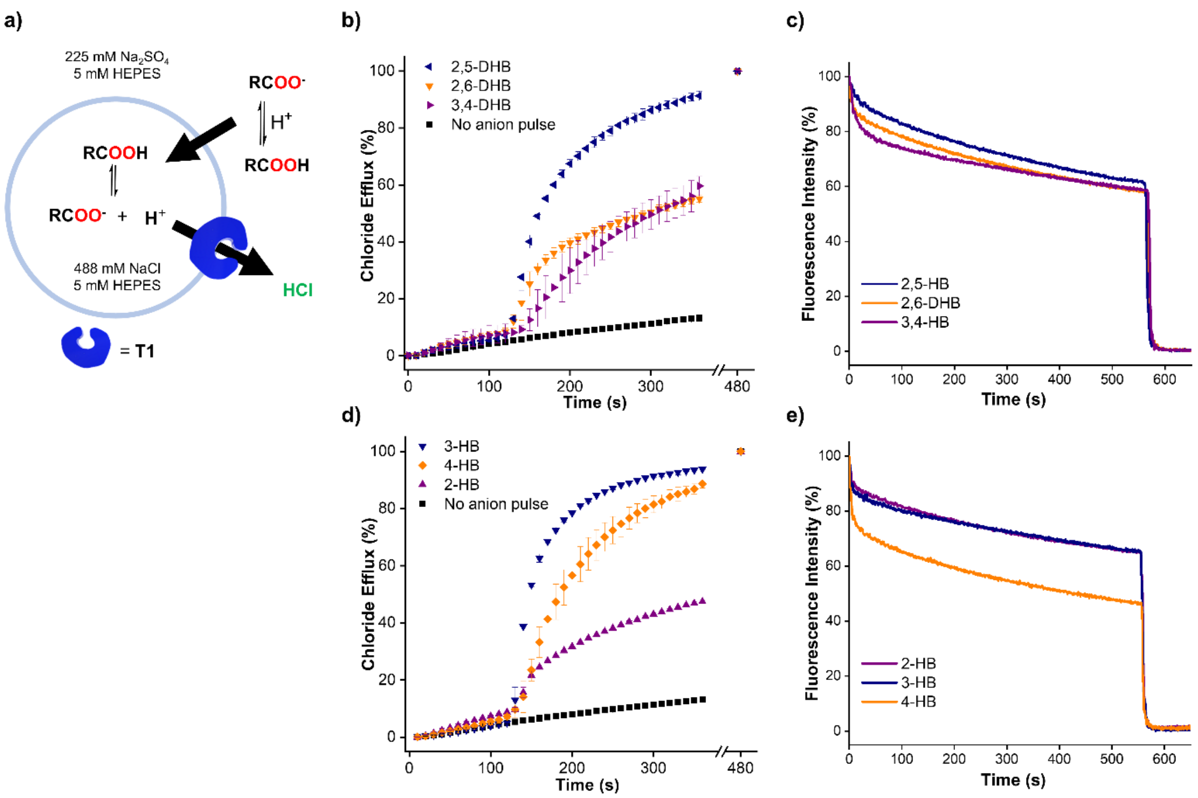

Figure S32: a) Potential mechanism for the chloride efflux generated by the spontaneous diffusion of carboxylic acids followed by the HCl release facilitated for the **T1** transporter. b) Transport (ISE-assay) and c) Diffusion (lucigenin assay) measured for selected DHB isomers. d) Transport and e) Diffusion measured for selected HB isomers.
